# Design, synthesis, and performance evaluation of TiO_2_-dye sensitized solar cells using 2,2′-bithiophene-based co-sensitizers

**DOI:** 10.1038/s41598-023-40830-1

**Published:** 2023-08-24

**Authors:** Mohamed R. Elmorsy, Fatma H. Abdelhamed, Safa A. Badawy, Ehab Abdel-Latif, Ayman A. Abdel-Shafi, Mohamed A. Ismail

**Affiliations:** 1 Department of Chemistry, Faculty of Science, Mansoura University, El-Gomhoria Street, Mansoura, 35516 Egypt; 2 Department of Chemistry, Faculty of Science, Ain Shams University, Abbassia, Cairo 11566 Egypt

**Keywords:** Chemistry, Optics and photonics

## Abstract

We report on the synthesis and characterization of six novel 2,2′-bithiophene-based organic compounds (**3a–c** and **5a–c**) that are designed to serve as co-sensitizers for dye-sensitized solar cells (DSSCs) based on TiO_2_. The compounds are linked to various donor and acceptor groups, and we confirm their chemical structures through spectral analyses. Our focus is on enhancing the performance of metal based **N3**, and the compounds were designed to operate at the nanoscale. We performed absorption and fluorescence emission measurements in dimethylformamide (DMF), where one of our compounds **5a** exhibited the longest maximum absorption and maximum emission wavelengths, indicating the significant impact of the para methoxy group as a strong electron-donating group. Our dyes **5a + N3** (η = 7.42%) and **5c + N3** (η = 6.57%) outperformed **N3** (η = 6.16%) alone, where the values of short current density (*J*_*SC*_) and open circuit voltage (*V*_*OC*_) for these two systems also improved. We also investigated the charge transfer resistance at the TiO_2_/dye/electrolyte interface using electrochemical impedance spectroscopy (EIS), which is important in the context of nanotechnology. According to the Nyquist plot, the **5a + N3** cocktail exhibited the lowest recombination rate, resulting in the highest *V*_*OC*_. Our theoretical calculations based on density functional theory (DFT) are also in agreement with the experimental process. These findings suggest that our compounds have great potential as efficient DSSC co-sensitizers. This study provides valuable insights into the design and synthesis of new organic compounds for use as co-sensitizers in DSSCs based on TiO_2_ and highlights the potential of these compounds for use in efficient solar energy conversion.

## Introduction

Solar cells manufactured using organic dyes, which are known as dye-sensitized solar cells (DSSCs), are a technology that falls under future technologies for the manufacture of solar cells at a low cost^1–4^. One of the essential principles in creating a dye-sensitized solar cell involves the creation of a highly porous nanocrystalline TiO_2_ layer^5^. This layer serves as the surface on which a photosensitizer, or dye, with a high molar extinction coefficient is chemically attached to form the working electrode of the solar cell. The working electrode is then separated from a platinum counter-electrode by an iodide-triiodide liquid electrolyte^6,7^. The electrolyte contains a redox couple, as $I_{3}^{ - } / I^{ - }$, closed by a counter electrode (often platinum)^8^.

Photosensitizers are a crucial component of DSSCs as they have the ability to convert incident light into excited electrons that can be used to generate electrical current. This makes their role critical in the overall performance of the DSSC, compared to other components^9^. There are two types of dyes that have shown efficiency when used in this application. The first type, which is a metal-free organic dye, is characterized by its high absorption strength and depends on the use of donor moieties such as phenothiazine, indoline, carbazole, triphenylamine and natural dye such as Betalain and Anthocyanin, extracted from beetroot and cranberries, or a blend of three natural photosensitizers derived from Roselle, spinach, and beetroot^10^, to improve the efficiency of DSSCs^11–13^, linker to the acceptor moieties diketopyrrolopyrrole, benzothiadiazole, cyanoacetamides, and benzotriazole^14–18^. The second type is metal-based dyes, the most famous of which are ruthenium compounds such as cis-Bis(isothiocyanato) bis(2,2′-bipyridyl-4,4′-dicarboxylato ruthenium(II) N3, di-tetrabutylammonium cis-bis(isothiocyanato)bis(2,2′-bipyridyl-4,4′-dicarboxylato)ruthenium(II) N719, and black dye^19,20^. The performance of the second type is better than the first, but it is disadvantaged by its high cost and complex preparation methods^21–23^. In order to take advantage of both types, the co-sensitization process, which is the use of different types of p; dyes in the same preparation, was used in this application^24–28^.

**Figure 1:**
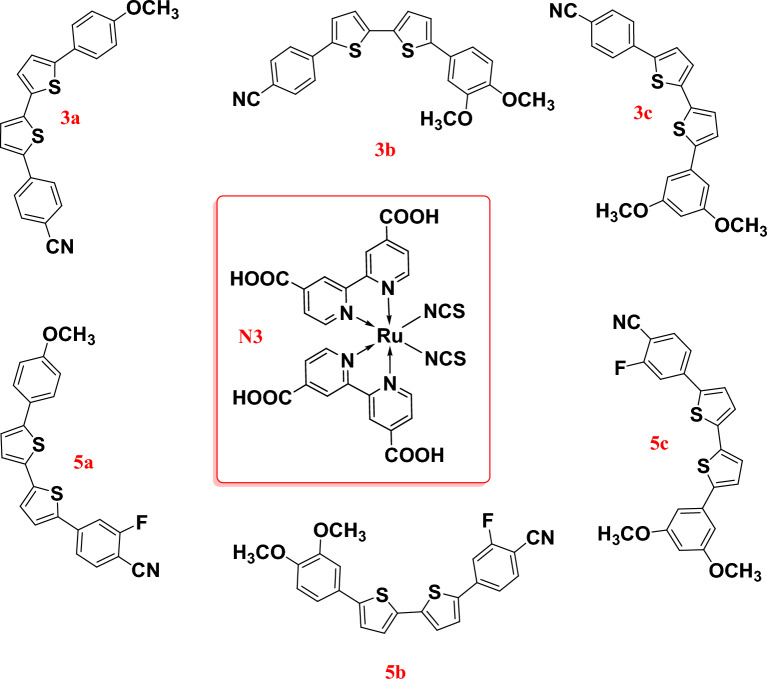
Molecular structures of co-sensitizers **3a–c, 5a–c** and **N3**^19^.

Our research contributes to the growing body of knowledge on the development of efficient and low-cost DSSCs, which have significant implications for renewable energy. The use of organic compounds as co-sensitizers in DSSCs is an active area of research, and our findings have the potential to advance this field further. Overall, our research highlights the importance of developing innovative approaches to improve the performance of DSSCs, ultimately contributing to the global effort to transition towards sustainable and clean energy sources. Therefore, based on what has been mentioned, organic compounds (**3a–c** and **5a–c**) that are easy to prepare and small in size have been prepared to be used as co-sensitizers to improve the commercial dye **N3**. The chemical composition of the different dyes and the commercial dye **N3** is shown in Fig. 1.

## Results and discussions

### Chemistry

**Figure 2:**
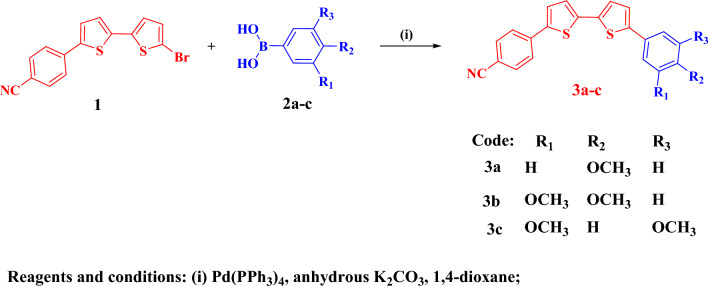
Synthesis scheme for the bithienylbenzonitrile derivatives.

The synthetic procedures for compounds **3a–c** and **5a–c** are provided in the supplementary file. The bithienylbenzonitrile derivatives **3a–c** were prepared via refluxing bromo bithienyl derivative **1**^29^ with 4-methoxyphenylboronic acid (**2a**), 3,4-dimethoxyphenylboronic acid (**2b**), and 3,5-dimethoxyphenylboronic acid (**2c**) in 1,4-dioxane as a solvent using Pd(PPh_3_)_4_ as a catalyst, and K_2_CO_3_ as a base (Fig. 2). Compound **3a** is prepared with updated procedure by Suzuki coupling reaction with good yield (75%) compared to 51% of the previous work^30^.

**Figure 3:**
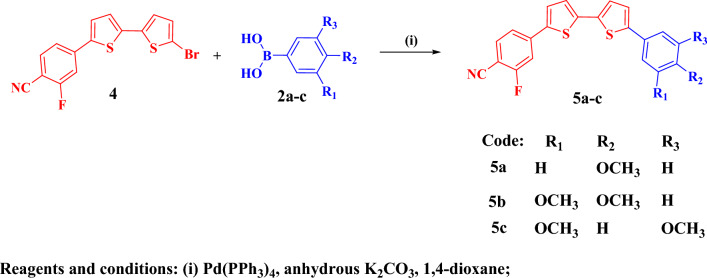
Synthesis scheme for the bithienylfluorobenzonitrile derivatives **5a–c**.

**Figure 4:**
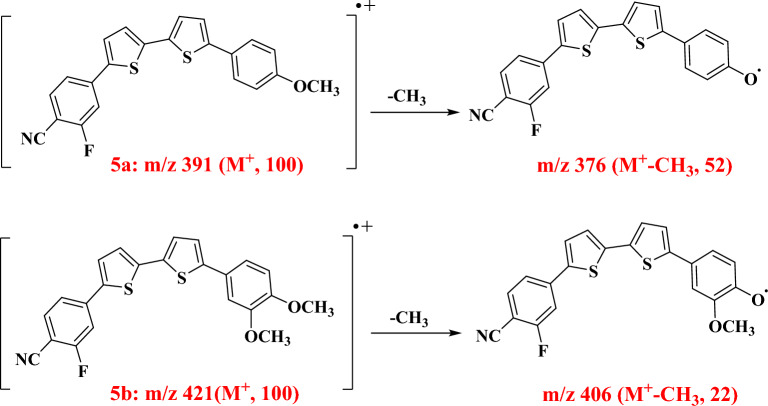
Mass fragmentation patterns of bithienylfluorobenzonitriles **5a, 5b**.

**Figure 5:**
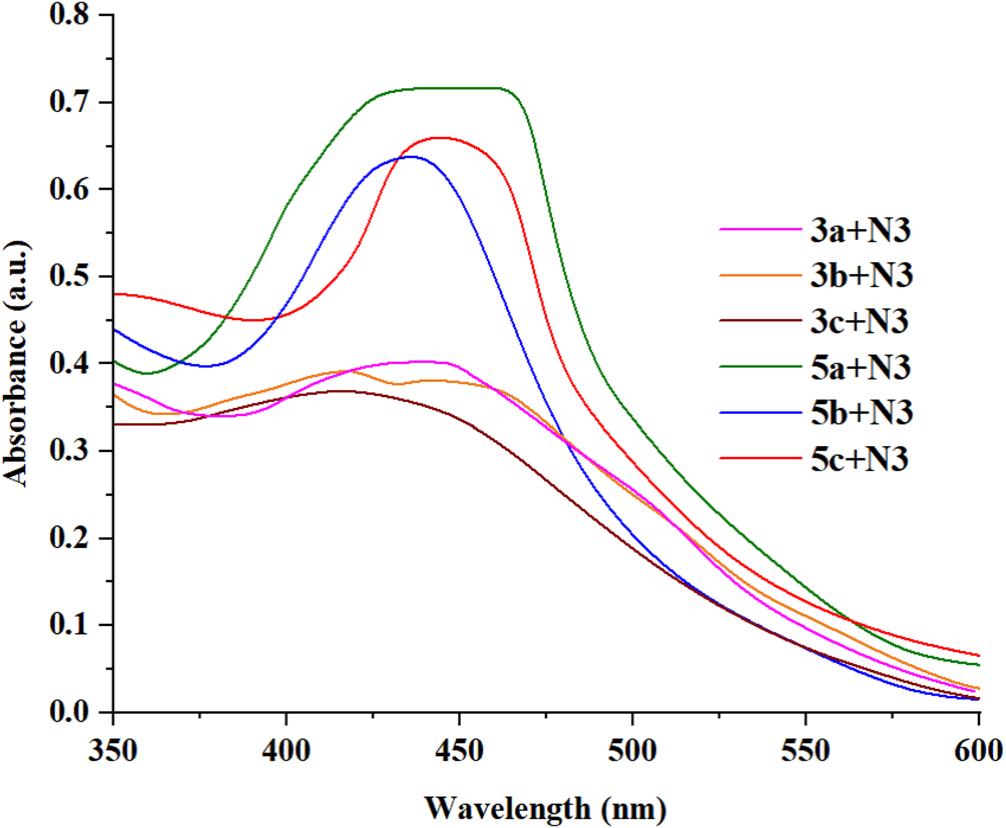
UV/Vis absorption of bithiophene dyes **3a–c** and **5a–c** absorbed over TiO_2_.

**Figure 6:**
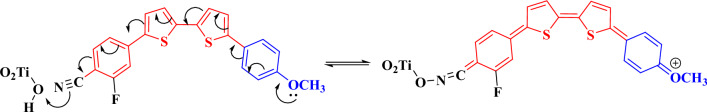
Molecular structure of **5a** binding to the titania surface.

**Figure 7:**
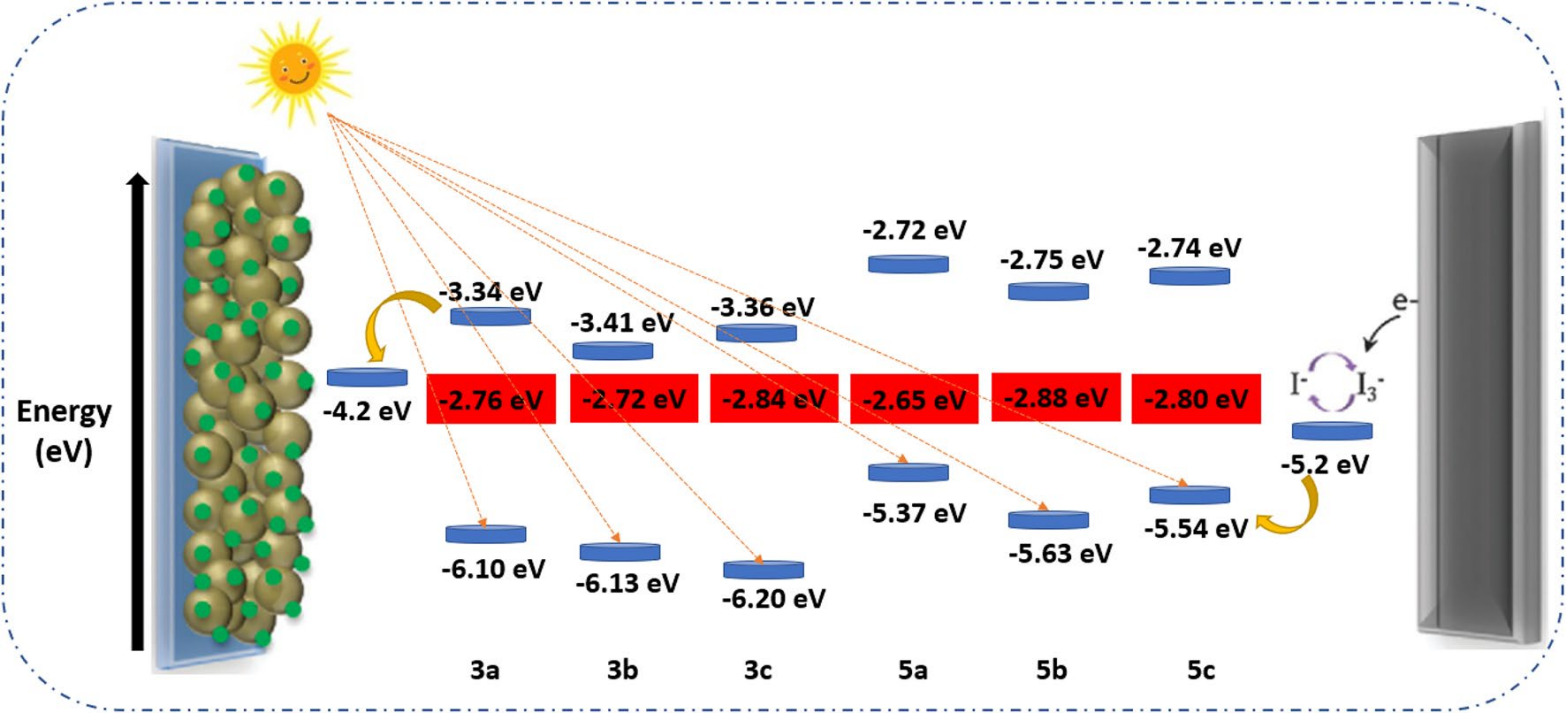
Energy-level diagram of bithiophene dyes **3a–c** and **5a–c**.

**Figure 8:**
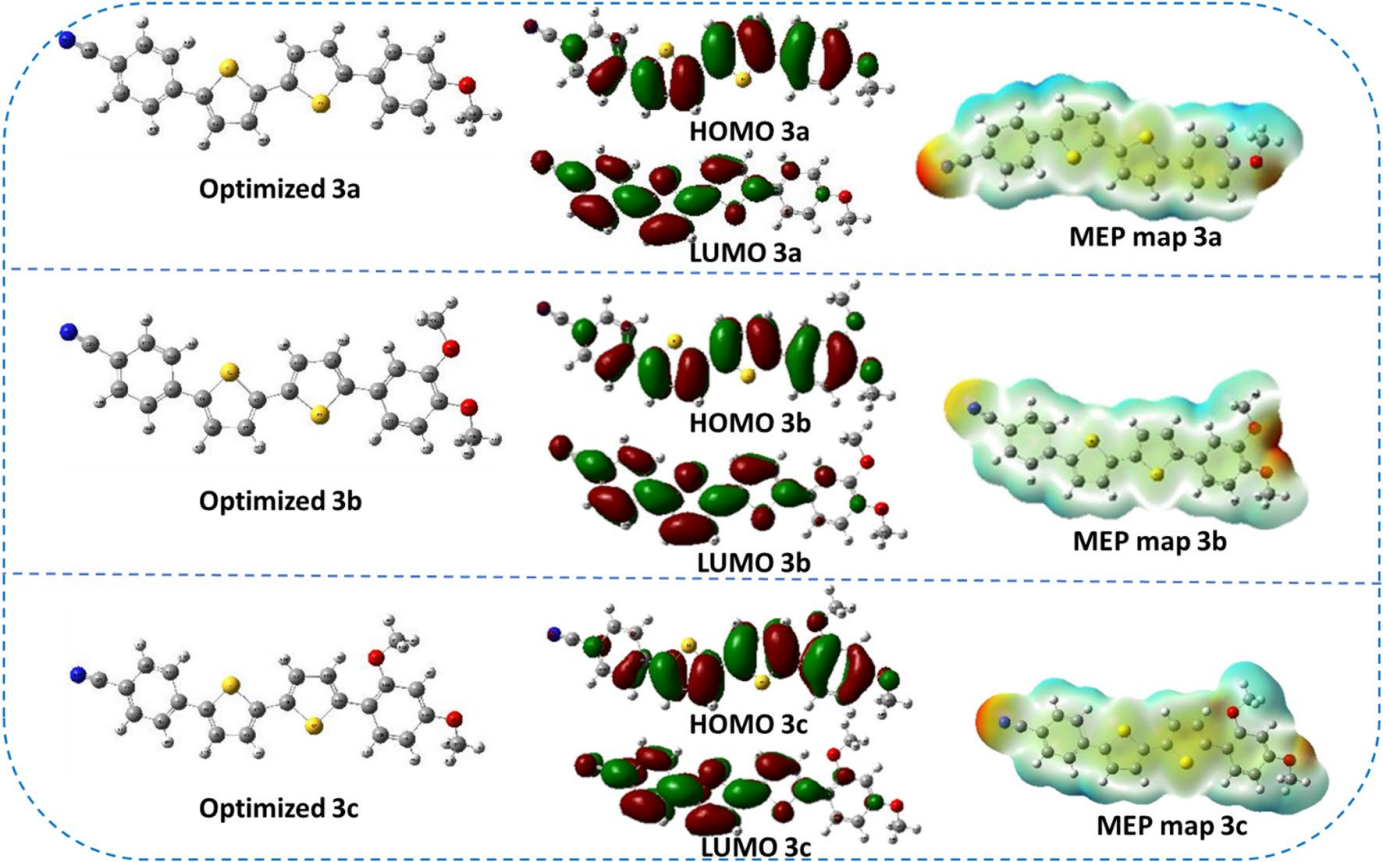
The optimized structures, FMOS and MEP, of co-sensitizers **3a–c**.

**Figure 9:**
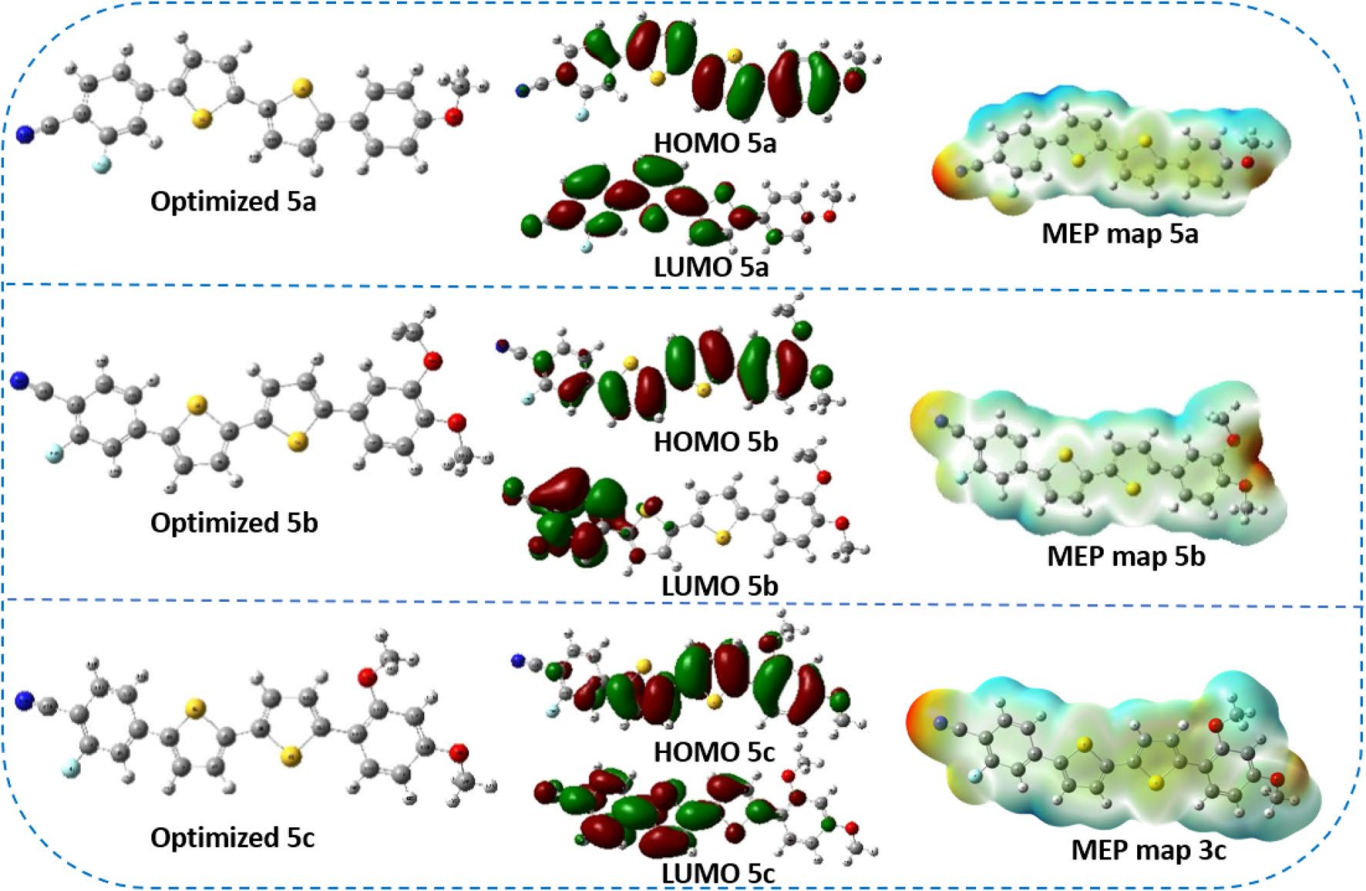
The optimized structures, FMOS and MEP, of co-sensitizers **5a–b**.

The elemental and spectral analyses were used to confirm the structures of bithienylbenzonitrile derivatives **3a-c**. The corresponding figures (Figures 1–9) are provided in the supplementary files. ^1^H-NMR spectrum of compound **3b** displayed two singlet signals of dimethoxy groups at δ 3.78 (*para* -OCH_3_), 3.83 (*meta* OCH_3_), two doublet signals, at δ 6.99 (*J* = 8.5 Hz), δ 7.22 (*J* = 2.5 Hz), along with doublet of doublet signal at δ 7.20 (*J* = 8.5, 2.5 Hz) corresponding for 1,3,4-trisubstituted benzene ring, and four signals as doublet referring to bithiophene-H’s at δ 7.40 (1H), 7.41 (1H), 7.46 (1H), and 7.74 (1H). In addition to, singlet signal at δ 7.86 (4H) of benzonitrile-H’s. Mass spectrum of compound **3b** gave a molecular ion peak m/z at 403 (M^+^, 100), along with a fragment at 388 (M^+^-CH_3_).

**Figure 10:**
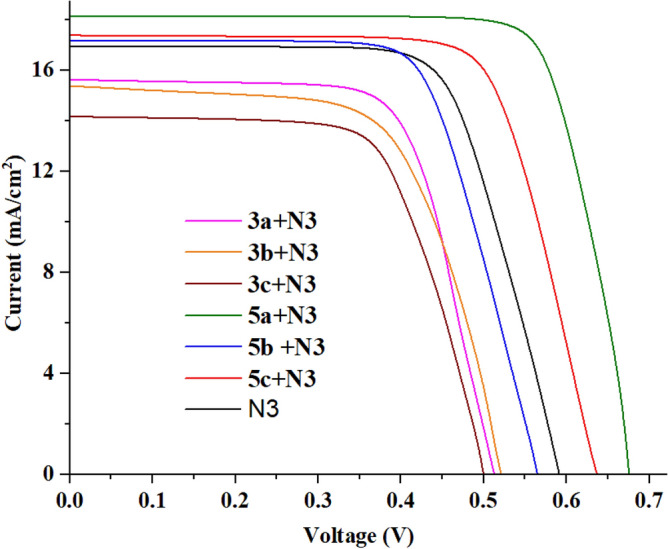
*I-V* characteristics curves of co-sensitizers **3a–b**, and **5a–c** with **N3**.

**Figure 11:**
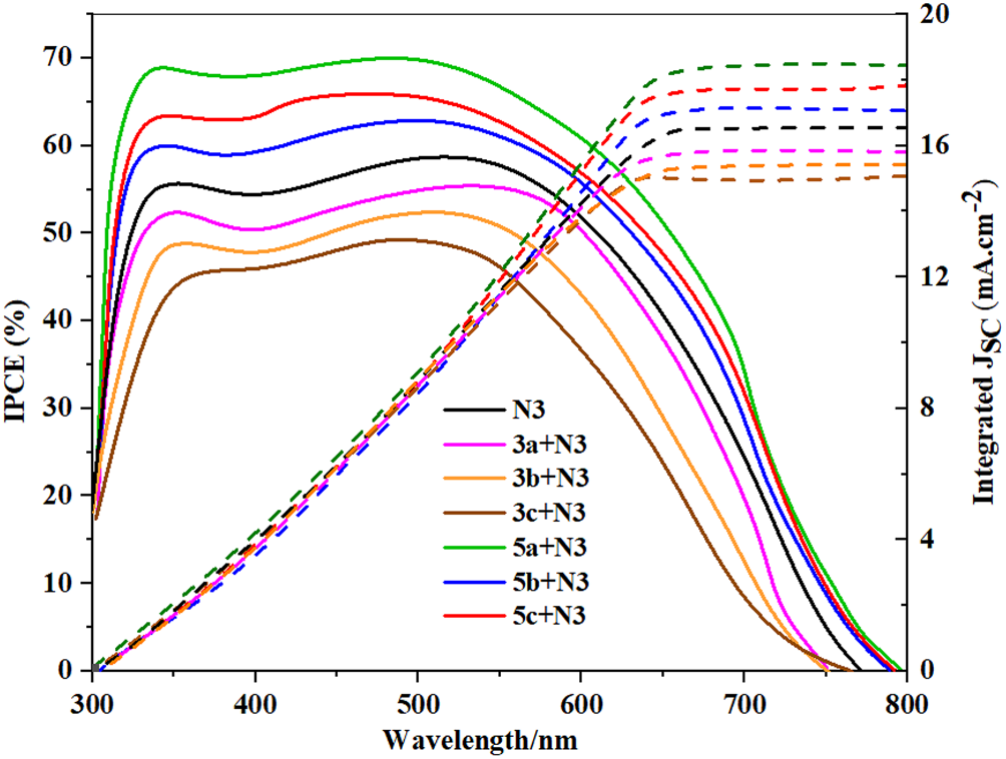
IPCE spectra and integrated currents of DSSCs based on co-sensitizers **3a–b**, and **5a–c** with **N3**.

**Figure 12:**
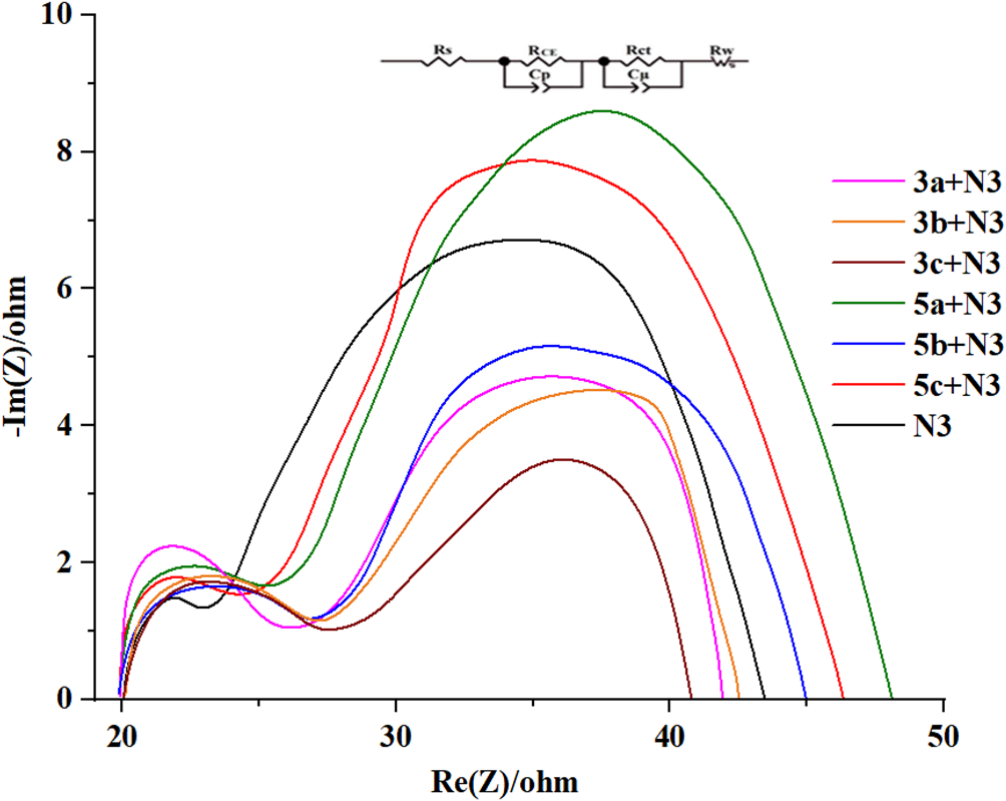
**Nyquist** plots of compounds **3a–c** and **5a–c** with **N3** based.

**Figure 13:**
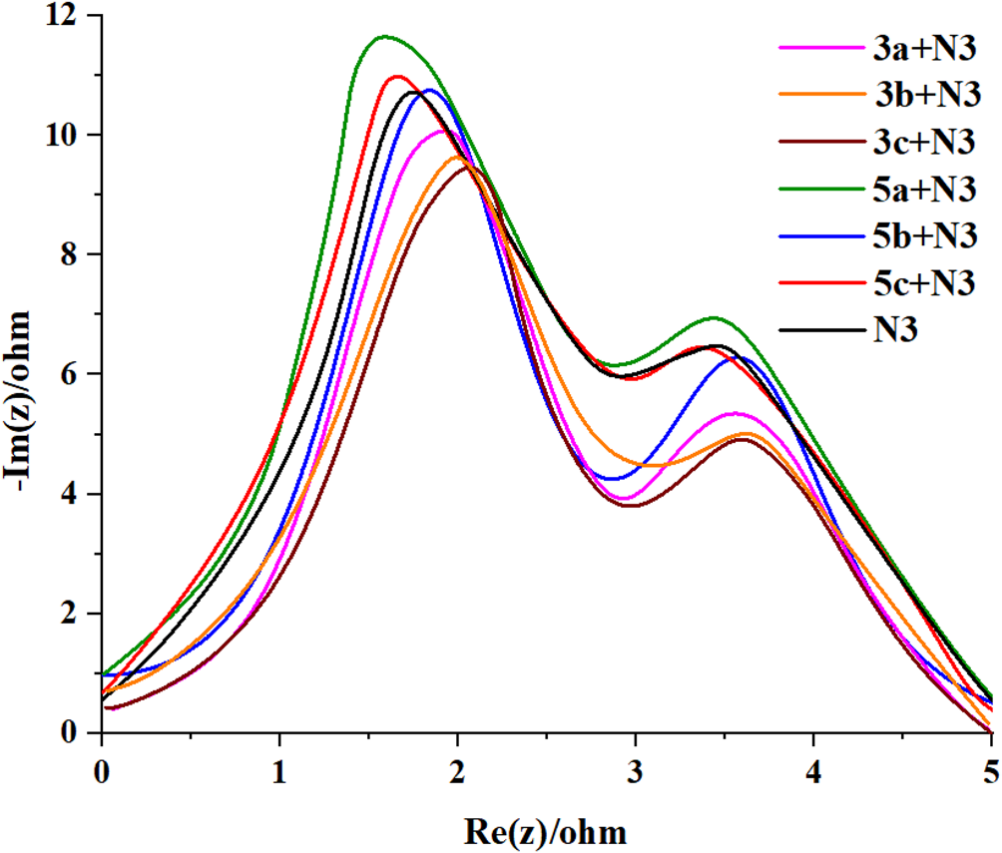
EIS Bode plots of compounds **3a–c** and **5a–c** with **N3** based.

**Figure 14:**
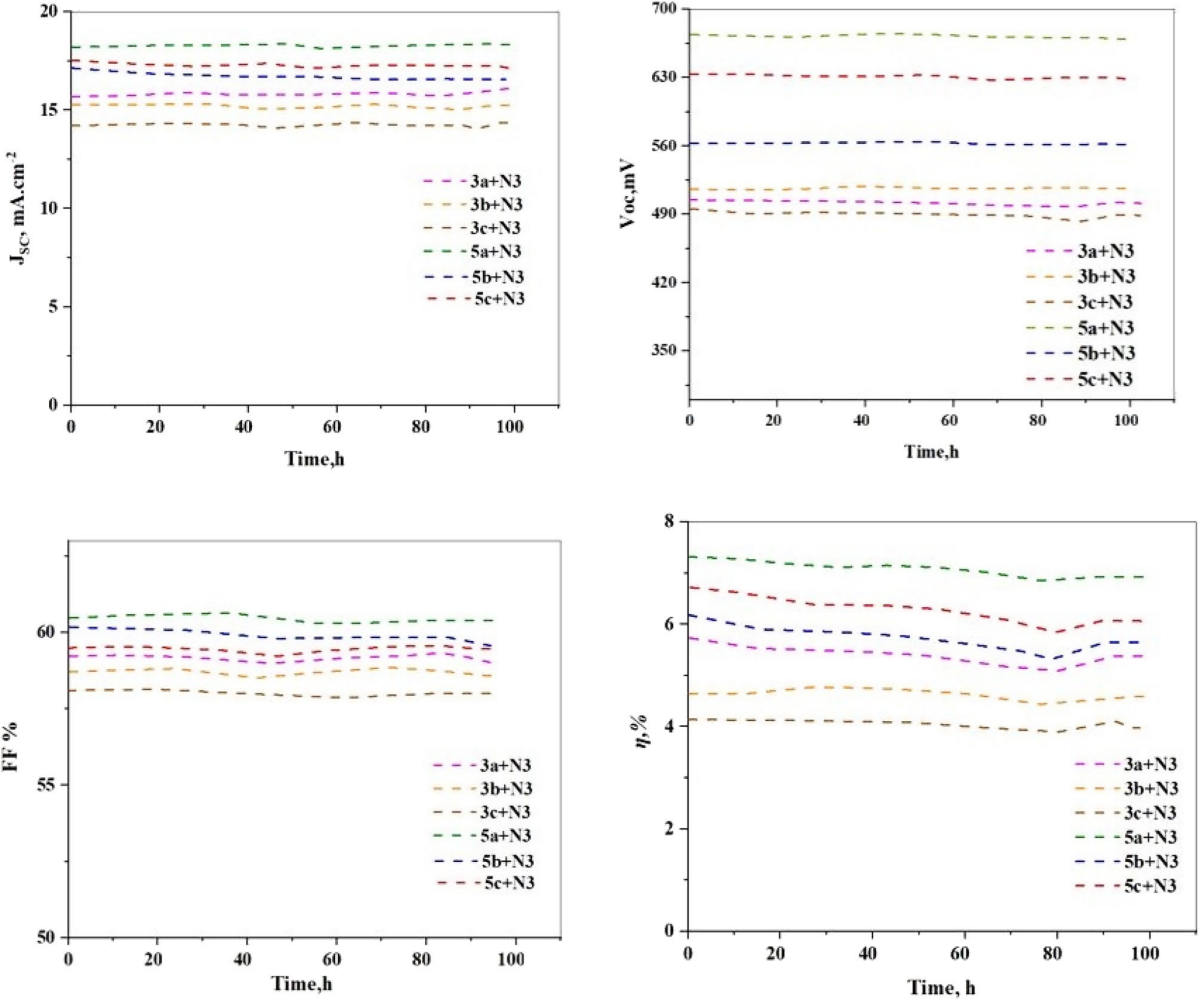
Stability of **3a–c** and **5a–c** with **N3** based.

Figure 3 outlines the preparation of the bithienylfluorobenzonitrile derivatives **5a–c** starting with a Suzuki coupling reaction via cross-coupling reaction of bromo bithienyl derivative **4**^31^ with the proper phenylboronic acids **2a–c**. Structures of bithienylfluorobenzonitriles **5a–c** were confirmed based on their elemental and spectral analyses. The corresponding figures (Figures 10–18) are provided in the supplementary files. IR spectra of the bithienyl fluorobenzonitriles **5a-c** indicated the presence of cyano group with stretching vibrations in the range of 2228–2229 cm^−1^. ^1^H-NMR spectrum of bithienyl flurobenzonitrile derivative **5a** displayed singlet signal referring to *para* -OCH_3_ at δ 3.78, AA’BB’ splitting pattern of two doublet signals at δ 6.98, 7.61 (*J* = 8.5 Hz, two protons each) of Ar–H of 4-methoxyphenyl ring and two doublet signals one proton each (*J* = 3.5 Hz) of one thiophene moiety and multiplet signal referring to 2H of the other thiophene moiety, along with doublet of doublet at δ 7.67 (*J* = 8.0, 1.5 Hz) and multiplet signal corresponding for 1,3,4-trisubstituted benzene ring. Moreover, mass spectrum of compound **5a** gave a molecular ion peak m/z at 391 (M^+^, 100) as a base peak, along with a fragment at 376 (M^+^-CH_3_) as outlined in Fig. 4.

^1^H-NMR spectrum of compound **5b** showed two singlet signals referring to 3,4-dimethoxy groups at δ 3.78 (*para* -OCH_3_), 3.83 (*meta* -OCH_3_), two doublet signals, at δ 6.99 (*J* = 8.0 Hz), δ 7.25 (*J* = 1.5 Hz), along with doublet of doublet at δ 7.22 (*J* = 8.0, 1.5 Hz) corresponding for 1,3,4-trisubstituted benzene ring, and four signals referring to bithiophene-H’s; in addition to, two signals integrated for Ar–H’s of fluorobenzonitrile ring. Mass spectrum of compound **5b** gave a molecular ion peak m/z at 421 (M^+^, 100) as a base peak, along with a fragment at 406 (M^+^-CH_3_) due to loss of a methyl group, mass fragmentation patterns (Fig. 4) were in consistent with the reported literature^32^.

### Optical measurements

The absorption and fluorescence emission measurements were performed in DMF. The bathochromic shift observed in the fluorescence emission spectra is not as pronounced as that observed in the corresponding absorption spectra. The wavelength of maximum absorption and *E*_*0–0*_ transition of the fluorescence emission spectra are collected in Table 1.

**Table 1:** Photophysical parameters of bithiophene derivatives **3a–c** and **5a–c**.

Co-sensitizers	λ_abs_ (nm)	λ_em_ (nm)	ν_abs_ (cm^−1^)	ν_em_ (cm^−1^)	*E*_*0–0*_^a^ (cm^−1^)	*E*_*0–0*_ (eV)
3a	401	512	24,937.66	19,531.25	22,234.45	2.7567
5a	415	537	24,096.39	18,621.97	21,359.18	2.6482
3b	404	525	24,752.48	19,047.62	21,900.05	2.71526
5b	381	496	26,246.72	20,161.29	23,204.00	2.8769
3c	395	489	25,316.46	20,449.9	22,883.18	2.8371
5c	392	507	25,510.2	19,723.87	22,617.03	2.80415

^a^Calculated as (ν_abs_ + ν_em_)/2.

As shown in Figure 19 in supplementary file presents a simple class of D–π–A that clearly shows the structural influence of the electron donor and electron acceptor groups. Bithiophene derivative **5a** shows the maximum absorbance ${\uplambda }_{{{\text{max}}}}^{{{\text{abs}}}}$ and maximum a emission ${\uplambda }_{{{\text{max}}}}^{{{\text{em}}}}$ indicating the effect of methoxy group as strong electron donating group and enhanced strength of the electron withdrawing cyano group by the presence of fluorine atom in its ortho position. Bithiophene derivatives **3c** and **5c** are similar in the electron donating *meta-*dimethoxy groups which approximately shows same fluorescence emission maximum and few nanometres bathochromic shift with the fluorinated analogue. The fluorinated analogue of the *ortho*-dimethoxy derivatives were found to enhance the bathochromic shift as has been observed for the *para*-mono methoxy analogue. This can be attributed to the presence of ICT process from the meta methoxy in the electron donating moiety to the meta fluorine atom in the acceptor group which interfere with the ICT from ortho methoxy to the cyano pathway. The absorption spectrum of **N3** is displayed at Figure 20 in the supplementary file.

We also studied the UV–Vis spectrum of the dyes on the surface of the TiO_2_ (Fig. 5). Through the results, it became clear that the spectrum is broader than that of the solution. This confirms the absorption of the dyes on the surface of the TiO_2_. It was also observed that the dyes **5a–c** had an absorption at a longer wavelength than that in the solution, which resulted from the *J*-aggregation^33^. The presence of two linkage groups (CN and F) results in the highest absorption of dyes **5a–c**, whereas the cyano group is present alone in dyes **3a–c**. The aforementioned results indicate that dyes **5a–c** could give interesting results when used in DSSCs. The absorption spectrum of **N3** anchored to TiO_2_ is displayed at Figure 21 in the supplementary file.

Figure 6 provides additional insight into the mechanism by which our nitrile dyes are anchored to the TiO_2_ surface. This anchoring process is supported by the fact that various compounds, including transition metal cyanides, have been observed to form visible charge-transfer (CT) complexes with Ti(IV) ions on surfaces^34–36^. This results in strong dye-to-TiO_2_ charge-transfer (DTCT) bands, indicating that the nitrile group-containing compounds, (**3a–c** and **5a–c**), serve as effective anchoring groups for TiO_2_.

The nitrile dyes can undergo one-step electron injection to the conduction band (CB) of TiO_2_ in a DSSC, similar to transition metal cyanides. Therefore, developing dye systems for electron injection to TiO_2_ through this pathway is of great interest academically and practically^36–40^. In this study, we focused on sensitizers with nitrile units that bind to the surface of TiO_2_ through chelation of surface Ti(IV) ions^41^. Among the nitrile sensitizers, sensitizer 5a, which contains an electron-withdrawing group (F) and an electron-donating methoxy group, is the best model. The dipolar structure of 5a provides directionality to electronic orbitals in the excited state, which improves direct, "one-step" electron injection from the ground state of the dye to the CB of TiO_2_ via photoinduced charge-transfer excitation of the dye-to-TiO_2_ bands^42^. The acidic hydrogen relocates to the titanium complex during the shift to the titanium complex from the dyes Figure 22 in the supplementary file. The orbital distribution and energy levels of each dye following the interaction with the TiO_2_ surface were investigated to study the electronic connection between the LUMO of the dye and the conduction band CB of the semiconductor.

### Electrochemical measurements

There are some requirements that must be met in order to use organic dyes in the field of DSSCs, which depend on the values of the ground state oxidation potential (GSOP) and excited state oxidation potential (ESOP)^34^. With the help of both the cyclic voltammetry and the UV–Vis spectrometer measurements, these values were calculated by the following equation^43^:

$$ {\text{ESOP}} = [{ }\left( {\left( {{\text{GSOP }}\left( {\text{V}} \right) + 4.7} \right) - E_{0 - 0} } \right]{\text{eV}} $$

From Fig. 7, It was found that the ESOP values of the bithiophene dyes **3a–c** and **5a–c** are categorical (− 3.34 eV, − 3.41 eV, − 3.36 eV, − 2.72 eV, − 2.75 eV, and − 2.74 eV) in order. Since it is higher than the value of the conduction band of the TiO_2_ (− 4.2 eV), the electrons will automatically flow easily to the TiO_2_^35^. On the other hand, the GSOP values for dyes were found as follows: − 6.10 eV (**3a**), − 6.13 eV (**3b**), − 6.20 eV (**3c**), − 5.37 eV (**5a**), − 5.63 eV (**5b**), and − 5.54 eV (**5c**). It was found at a lower level than the electrolyte (− 5.2 eV), which helps return electrons to the dye to start over^44–46^. All this means that the dyes met the conditions for their use in DSSCs. The **5a–c** dyes achieved LUMO values at higher levels than the **3a–c** dyes, which means that the flow rate of electrons in these dyes to the surface of the semiconductor is higher, and this also indicates that the dyes will give high efficiency when used in DSSCs.

### Theoretical investigation

Theoretical calculations using the density functional theory (DFT) of the Gaussian program were applied to delve deeper into the structural composition of the compounds under study. B3LYP/6-311G (d, p) was used as the functional model and basis set for the first optimization of the structures^47–49^. The values of GSOP, ESOP, *E*_*0–0*_, and simulated frontier molecular orbitals (FMOs) are displayed and calculated for the different affirmations, and they proved to be in agreement with the practical values that we obtained (Table 2).

**Table 2:** Electrochemical data for compounds **3a–c** and **5a–c**.

Compound	Practical results (eV)			Theoretical calculations (eV)		
	*E*_*0–0*_	GSOP	ESOP	*E*_*0–0*_	GSOP	ESOP
**3a**	2.76	− 6.10	− 3.34	2.73	− 5.92	− 3.19
**3b**	2.72	− 6.13	− 3.41	2.68	− 5.96	− 3.28
**3c**	2.84	− 6.20	− 3.36	2.65	− 5.99	− 3.34
**5a**	2.65	− 5.37	− 2.72	2.61	− 5.31	− 2.70
**5b**	2.88	− 5.63	− 2.75	2.81	− 5.56	− 2.75
**5c**	2.80	− 5.54	− 2.74	2.79	− 5.48	− 2.69

Figure 8 shows the optimized structures, FMOS and MEP, of co-sensitizers **3a–c**. From their FMOS, the distribution of the electrons in the HOMO is concentrated on the whole molecule, whereas in the LUMO, there was a shift to the acceptor part toward the cyano-moiety. The computed contributions of various electronic transitions, presented in Table 1 (supplementary file), suggest that the ICT in co-sensitizers 3a-c and 5a-c is characterized by a peak absorption in the 470–478 nm range, related to the large contribution of HOMO to LUMO transitions Meanwhile, the π–π* electronic transitions of the compounds, due to significant contributions of HOMO-n to LUMO + n transitions^50^. Using the same basis set and functional energy, we calculated the electrostatic potentials (MEPs) of the dyes under investigation. The MEP concentrates on anticipating physicochemical features like the reactive groups associated with the molecular structure^51^. Also, MEP is a graph of the electrostatic potential, plotted onto the constant electron density surface^52^. Different colors are used to show ESP levels. An electron-rich field is shown by the red area, while electron-poor sections are indicated by the blue region^53^.

Also, Fig. 9 shows the optimized structures, FMOS and MEP, of co-sensitizers **5a–c**. For co-sensitizers **5a–c**, the distribution of the electrons in the HOMO is concentrated on the donor part, whereas in the LUMO, there was a shift to the acceptor part toward the acceptor-anchoring moiety, which indicated better charge transfer as shown in their FMO levels. MEP also demonstrated the high electron density parts and low charge parts according to their different colors, from red to blue.

### Photovoltaic measurements:

With an iodolyte electrolyte and simulated AM 1.5 G illumination, photocurrent density–voltage (*I–V*) curves of DSCs based on dyes **3a–b** and **5a–b** together with **N3** were done. The supplementary file contains information on the fabrication process and instruments used for the photovoltaic measurements^54–56^. Because of what the previous measurements showed and the small size of the dyes **3a–c** and **5a–c** that were made, they were used as co-sensitizers in DSSCs to make the standard dye (**N3**) work better. Figure 10 displays the *I–V* curves of the co-sensitizers **3a–c**, **5a–c** and **N3**. The corresponding data is recorded in Table 3.

**Table 3:** Photovoltaic parameters for co-sensitizers **3a–c**, **5a–c** and **N3**.

Dye	*V*_*OC*_^a^ (*V*_*OC*_^b^)/V	*J*_*SC*_^a^ (*J*_*SC*_^b^) (mA cm^−2^)	*FF*^a^ (*FF*^b^)/%	*η*^a^ (*η*^b^)/%	Concentration of the dye/10^–5^ mol cm^−2^)
3a + N3	**0.511** (0.5 ± 0.010)	**15.67** (15.26 ± 0.37)	**59.16** (59.02 ± 0.13)	**4.73** (4.47 ± 0.24)	**0.88**
3b + N3	**0.519** (0.488 ± 0.028)	**15.38** (14.93 ± 0.44)	**58.13** (57.91 ± 0.27)	**4.64** (4.22 ± 0.32)	**0.75**
3c + N3	**0.499** (0.458 ± 0.048)	**14.13** (0.13.55 ± 0.53)	**58.55** (58.16 ± 0.51)	**4.13** (3.62 ± 0.55)	**0.61**
5a + N3	**0.676** (0.625 ± 0.041)	**18.14** (17.82 ± 0.37)	**60.54** (60.18 ± 0.29)	**7.42** (6.71 ± 0.56)	**2.65**
5b + N3	**0.565** (0.531 ± 0.042)	**17.17** (16.80 ± 0.33)	**60.01** (58.95 ± 0.09)	**5.82** (5.38 ± 0.44)	**1.54**
5c + N3	**0.637** (0.591 ± 0.041)	**17.42** (17.14 ± 0.23)	**59.18** (58.77 ± 0.51)	**6.57** (5.96 ± 0.53)	**1.98**
N3	**0.590** (0.564 ± 0.034)	**16.92** (16.71 ± 0.23)	**62.09** (61.73 ± 0.48)	**6.28** (5.85 ± 0.42)	**1.19**

Significance values are in bold.

^a^The best device parameters (listed in the manuscript).

^b^The average device parameters (obtained from three devices).

As shown in Table 3, dyes **5a + N3** (*η* = 7.42%) and **5c + N3** (*η* = 6.57%) achieved higher efficiencies than **N3** (*η* = 6.16%) alone. The value of *J*_*SC*_ and *V*_*OC*_ for these two systems also improved. The dye absorbs the main dye, increasing the number of electrons flowing into the conductor and resulting in a high *J*_*SC*_ value. As for the increase in *V*_*OC*_, it is due to the filling of the existing interstitial spaces due to the adsorption of the dyes, which led to a larger number of dyes that bore on the surface of the conductor, and this leads to a decrease in aggregation and an increase in the voltage. Also, the co-sensitization impact of **3a–c**, **5a–c**, and **N3** was further investigated by measuring the loading onto the photoanode, which directly proportional to the photocurrent value. Table 3 summarizes the findings of measuring the concentration of co-sensitizers **3a–c**, **5a–c**, and **N3** adsorbed on TiO_2_ using 0.1 M NaOH in a dimethylformamide and water (1:1) combination. As a result of its improved *J*_*SC*_ and suitably expanded light collecting capacities, **5a** provided the optimum co-sensitization performance. The increase in ***V***_***OC***_ and ***J***_***SC***_ consequently led to an increase in cell efficiency.

It is also noted that the dyes **3a–c** did not achieve a positive effect on the dye **N3**, and this was originally due to the nature of the co-sensitizer and its inability to adhere well to the surface of the TiO_2_, i.e., an aggregation of the dye occurred, and the recombination increased, so the voltage decreased. Also, the **N3** didn't get well absorbed because the **N3** and co-sensitizer were aggregated together. This made it harder for electrons to get into the TiO_2_, so the ***J***_***SC***_ went down. Table 2 (Supplementary file) compares the performance parameters of **3a–c**, **5a–c** and **N3** with recently reported high-performance sensitizer and co-sensitizer. Furthermore, the electronic distribution for co-sensitizers **3a–c**, from Fig. 8, the HOMO for co-sensitizers **3a–c** is randomly distributed throughout the framework, whereas the LUMOs were located in the anchoring parts (cyano units) through the biphenyl-spacer, this means that the LUMO electron destination does not really tightly coincide with TiO_2_’s conduction band (CB), which could lead to poor electron injection efficiency from the dyes' LUMO and so the ***J***_***SC***_ values went down comparing to the others co-sensitizers and increasing the charge recombination rate at the interface of the TiO_2_/dye/electrolyte, which can be related to the energy level alignment at the interface. The bithienyl co-sensitizers **5a–c** exhibit efficient charge transfer between their highest occupied molecular orbital (HOMO) and lowest unoccupied molecular orbital (LUMO) levels, as seen in Fig. 9. The electron density is primarily localized on the substituent anchoring groups, allowing for electronic interactions between the TiO_2_ d-orbitals and the LUMO electron density. Additionally, the coexistence of the HOMO and LUMO levels facilitates rapid photo-induced electron transfer from the donating portion to the acceptor group. Because of this increased charge separation, 5a has a much higher photovoltaic efficiency than other sensitizers. Further dye **5b** did not improve the performance of the **N3** although the increased in the ***J***_***SC***_, which may be attributed to the recombination of the dye on the surface that result in the decreased *V*_*OC*_ value and hence the decrease of the efficiency. To learn more about the mechanism of the absorption of dyes **3a–c** and **5a–c** on the surface of TiO_2_, the IR-spectrum of the dyes on the surface of TiO_2_ was measured, as shown in Figures 23–28 in the supplementary files. It was found that the value of the cyano group (CN) disappeared, or its absorption decreased in the samples, which confirms that the binding was through the cyano group. Figure 11 shows the incident photon-to-current conversion efficiency (*IPCE)* spectra of devices sensitized with **N3** and co-sensitized with 2,2′-bithiophene-based **3a–c and 5a–c**. The *IPCE* spectra for all these cells exhibited a broad response within a large wavelength range (300–650 nm), indicating that all the dyes can efficiently convert the visible light into photocurrent. The higher *IPCE* values of the devices co-sensitized with the bithiophene co-sensitizers **3a–c and 5a–c** can be attributed to their improved photovoltaic characteristics. Additionally, **N3**-based cells fabricated with co-sensitizer **5a–c** demonstrated higher quantum efficiency (*IPCE* value) than **N719** alone, possibly due to the appropriate mixing of organic dye and Ru-II complex and presence of auxiliary electron withdrawing group. Furthermore, the increased *IPCE* values can be attributed to increased *J*_*SC*_ values, the *IPCE* integral areas of DSSCs exhibit an order for dyes of **5a + N3 > 5c + N3 > 5b + N3 > N3 > 3a + N3 > 3b + N3 > 3c + N3**, trend of is consistent with the trend of *J*_*SC*_. The cell co-sensitized by **5a + N3** had the highest *IPCE* response corresponding to its highest *Jsc* value of 18.14 mA cm^−2^ and gave over 65% *IPCE* values from 300 to 650 nm. This observation strongly advocates that during the dye loading process of co-sensitizers along with **N3** should have interacted with each other. Such interaction generally tends to induce electron and energy transfer between the two kinds and hence causes deterioration in cell performance. The co-sensitized cell exhibits the highest *Jsc* value in the *J-V* measurements, which can be attributed to its broadest and highest *IPCE* response^57^. Additionally, the co-sensitized cell's improved *Voc* value, coupled with its highest *Jsc* value, contributes to its further increased *PCE* value when compared to that of **5a + N3,** the improved *IPCE* response of (3a-c, and 5a-c is interpreted in terms of higher *J*_*SC*_ value which showed the same order of **5a + N3** = 18.23 > **5c + N3** = 17.52 > **5b + N3** = 17.21 > **3a + N3** = 15.83 > **3b + N3** = 15.59 > **3c + N3** = 15.02 mA cm^−2^ compared to **N3** (16.93 mA cm^−2^). These results implies that the structural optimization with 3a–c and 5a–c architecture is a key in getting greater efficiency, compared to the *Jsc* values obtained from the *J–V* data, the *J*_*sc*_^*IPCE*^ values integrated from the *IPCE* spectra are quite consistent. As a result, the co-sensitizer **5a** dye produces the most abundant *IPCE* spectrum, proving that it also has the greatest *Jsc*. The enhanced *IPCE* replies match the enhanced *Jsc* results.

On the other hand, the value of cyano and F disappeared for **5a–c**, which confirms the association of these dyes with cyano and fluorine together. Electrochemical impedance spectroscopy (EIS) was used to study the charge transfer resistance at the TiO_2_/dye/electrolyte interface^58,59^. This was done to learn more about the relationship between the structure of the molecules and their photovoltaic performance. The middle area shown in the Nyquist plot, as shown in Fig. 12, represents the relationship between the TiO_2_, the dye, and the electrolyte. The diameter of this part of the curve has a clear relationship with dye recombination; the larger the diameter, the less likely it is to be recombined, resulting in an increase in open circuit voltage^60^. On the Nyquist plots, the radius of the large semicircle in the middle frequency range was found to be **5a + N3** > **5c + N3** > **N3** > **5b + N3** > **3b + N3** > **3a + N3** > **3c + N3**, which shows the order of charge recombination resistance *R*_*rec*_ at the TiO_2_/dye/electrolyte interface. DSSCs with an increasing *R*_*rec*_ value have slower charge recombination between the electron injected and $I_{3}^{ - }$ ions in the electrolyte. The charge recombination resistance of these dyes (*R*_*CT*_) corresponding to the diameter of the middle frequency semicircle was calculated to decrease in the order of **5a + N3** (23.21 Ω), **5c + N3** (21.24 Ω), **N3** (20.34 Ω), **5b + N3** (18.19 Ω), **3b + N3** (16.58 Ω), **3a + N3** (15.56 Ω), **3c + N3** (14.29 Ω), in good agreement with the order photovoltage data. As a result, the *V*_*oc*_ increases. Accordingly, this sequence of *V*_*oc*_ values appears to be consistent. As a result, the **5a + N3** system with the largest diameter has the lowest recombination rate and the highest *V*_*OC*_, which is consistent with the photovoltaic values.

In Bode frequency plots Fig. 13, by applying the following equation the electron lifetime for injected electrons into TiO_2_ conduction band can be calculated by (τ_eff_ = 1/2π*f*)^58^, where τ represents the electron lifetime injected into TiO_2_ and *f* is the mid-frequency peak in bode plots, which is directly related to the electron lifetimes. The electron lifetimes of DSSCs that were sensitized using co-sensitizers were determined through Bode frequency plots. By analyzing the Bode phase plots, the frequency of the peaks observed in the middle-frequency domain can be used to assess the electron lifetime within the semiconductor (***τ***_***r***_*** (ms***). This provides additional insight into the charge recombination rate occurring at the interface of TiO_2_/dye/electrolyte. The values of the mid-frequency peaks of the bode plots displayed the order: **5a + N3** > **5c + N3** > **N3** > **5b + N3** > **3b + N3** > **3a + N3** > **3c + N3,** indicating the corresponding electron lifetimes ranked as: **5a + N3** (4.38 ms) > **5c + N3** (3.56 ms) > **N3** (3.40 ms) > **5b + N3** (2.62 ms) > **3b + N3** (1.64 ms) > **3a + N3** (1.52 ms) > **3c + N3** (1.26 ms), also coincided well with *V*oc. The electron lifetime and charge recombination rate at the interface of TiO_2_/dye/electrolyte are affected by factors such as the size and shape of the dye molecule^54^, as well as the dye adsorption behavior. These factors have a strong influence on the photovoltage of solar cells, as has been previously reported in the literature. In this study, the values of *R*_*re*c_ and se for the new co-sensitizer dyes were found to be consistent with the corresponding *V*_*OC*_ values obtained for the solar cells. The use of the **5a** co-sensitizer in DSSCs resulted in higher *V*_*OC*_ values compared to other sensitizers, including **N3**, due to the lower charge recombination rate at the TiO_2_/dye/electrolyte interface, which is attributed to the strength of the donating moiety.

The stability of dye-sensitized solar cells (DSSCs) is a key issue that must be addressed to ensure their long-term viability as a renewable energy technology, the stability of co-sensitizers has been measured and added into the manuscript, As depicted in Fig. 14, the high photo-stability of the **3a–c** and **5a–c/N3** dyes for DSSC applications is confirmed by the fact that the cell exhibits excellent stability with no discernible degradation of the initial performances even after 100 h of illumination. The superior long-term stability of co-sensitizer **5a** may be attributed to the hydrophobic properties of the substituted methoxy group attached to bithienyl spacer addition to acceptors moieties (F and CN).

## Conclusion

Our team accomplished significant success by designing and synthesizing six organic dyes (**3a–c** and **5a–c**) using a simple yet effective D–π–A structure. By displaying the structural effects of electron donor and acceptor groups, these dyes have evaluated a new trend for improving the photovoltaic capabilities of dye-sensitized solar cells (DSSCs). To evaluate the efficiency of our dyes, we co-sensitized them with the standard dye **N3**. The absorption spectra of co-sensitized TiO_2_ films become more intense and broader than the individual dyes. The EIS data indicates a reduced recombination of injected electrons with the triiodide ions and a longer electron lifetime. Therefore, an improvement of open circuit photovoltage (*Voc*) is achieved for **5a and 5c**. Under optimal conditions, the power conversion efficiency ranges (4.13–7.42%) and short circuit current (*J*_*SC*_) range (14.13–18.14 mA cm^−2^). Our mixed dyes **5a** and **N3** exhibited a remarkable efficiency of 7.42%, and with further optimization, we obtained a short-circuit current density of 18.14 mA cm^−2^ and a high open-circuit voltage (*V*_*OC*_) of 0.676 V. The exceptional J_SC_ value can be attributed to the presence of two linkage groups (CN and F) that increase the absorption of dye **5a**, as confirmed by the UV–Vis absorption over TiO_2_ surfaces. The maximum V_OC_ value, on the other hand, is due to the extensive surface area covered by both dyes **5a** and **N3**. The increase in Jsc and *Voc* are strongly correlated to enhance light absorption and reduced charge recombination, respectively. Our findings suggest that tuning the electron-donating groups is critical to improving the light-harvesting efficiency of co-sensitizers.

### Supplementary Information

Supplementary Information.

## Data Availability

All data supporting the conclusions of this article are included within the article and supplementary document.

## References

[1] Panwar NL, Kaushik SC, Kothari S (2011). Role of renewable energy sources in environmental protection: A review. Renew. Sustain. Energy Rev..

[2] Grätzel M (2001). Photoelectrochemical cells. Nature.

[3] Arshad M, Arshad S, Janjhi FA, Khan MS, Tariq MA, Hassan MT, Mehboob MY (2023). In silico modeling and exploration of new acceptor molecules with enhanced power conversion efficiency for high-performance organic solar cell applications. J. Solid State Chem..

[4] Zheng P, Xu J, Peng F, Peng S, Liao J, Zhao H, Li L, Zengb X, Yu H (2021). Novel dual acceptor (D–D′–A′–π–A) dye-sensitized solar cells based on the triarylamine structure and benzothiadiazole double electron withdrawing unit. New J. Chem..

[5] Atilgan A, Yildiz A (2022). Ni-doped TiO_2_/TiO_2_ homojunction photoanodes for efficient dye-sensitized solar cells. Int. J. Energy Res..

[6] Mehboob MY, Hussain R, Irshad Z, Adnan M (2021). Role of acceptor guests in tuning optoelectronic properties of benzothiadiazole core based non-fullerene acceptors for high-performance bulk-heterojunction organic solar cells. J. Mol. Model..

[7] Birel Ö, Nadeem S, Duman H (2017). Porphyrin-based dye-sensitized solar cells (DSSCs): A review. J. Fluoresc..

[8] Das P, Ganguly S, Maity PP, Bose M, Mondal S, Dhara S, Das AK, Banerjee S, Das NC (2018). Waste chimney oil to nanolights: A low cost chemosensor for tracer metal detection in practical field and its polymer composite for multidimensional activity. J. Photochem. Photobiol. B Biol..

[9] Yildiz A (2021). Efficient iron phosphide catalyst as a counter electrode in dye-sensitized solar cells. ACS Appl. Energy Mater..

[10] Teja AS, Srivastava A, Sathrughna JAK, Tiwari MK, Kanwade A, Yadav SC, Shirage PM (2022). Optimal processing methodology for futuristic natural dye sensitized solar cells and novel applications. Dyes Pigm..

[11] Singh AK, Nithyanandhan J (2022). Indoline-based donor-π-acceptor visible-light responsive organic dyes for dye-sensitized solar cells: Co-sensitization with squaraine dye for panchromatic IPCE response. ACS Appl. Energy Mater..

[12] Elmorsy MR, Badawy SA, Salem KE, Fadda AA, Abdel-Latif E (2022). New photosensitizers that are based on carbazoles and have thiophene bridges with a low bandgap do 32% better than N719 metal complex dye. J. Photochem. Photobiol. A..

[13] Elmorsy MR, Badawy SA, Abdel-Latif E, Assiri MA, Ali TE (2023). Significant improvement of dye-sensitized solar cell performance using low-band-gap chromophores based on triphenylamine and carbazole as strong donors. Dyes Pigm..

[14] Souilah, M., Hachi, M., Fitri, A., Benjelloun, A. T., Benzakour, M., Mcharfi, M. & Zgou, H. Improved photovoltaic performances of coumarin derivatives by forming DA′-Π-A structure using diketopyrrolopyrrole as auxiliary acceptor. *SSRN* 4116061 (2022).

[15] Gonzalez-Flores CA, Pourjafari D, Escalante R, Canto-Aguilar EJ, Poot AV, Castán JMA, Kervella Y, Demadrille R, Riquelme AJ, Anta JA, Oskam G (2022). Influence of redox couple on the performance of ZnO dye solar cells and minimodules with benzothiadiazole-based photosensitizers. ACS Appl. Energy Mater..

[16] Badawy SA, Abdel-Latif E, Fadda AA, Elmorsy MR (2022). Synthesis of innovative triphenylamine-functionalized organic photosensitizers outperformed the benchmark dye N719 for high-efficiency dye-sensitized solar cells. Sci. Rep..

[17] Aldusi AM, Fadda AA, Ismail MA, Elmorsy MR (2022). Simple organic dyes containing multiple anchors as effective co-sensitizers for DSSCs loaded with Ru(II) complex N-719. Appl. Organomet. Chem..

[18] Elmorsy MR, Su R, Abdel-Latif E, Badawy SA, El-Shafei A, Fadda AA (2020). New cyanoacetanilides based dyes as effective co-sensitizers for DSSCs sensitized with ruthenium(II) complex (HD-2). J. Mater. Sci. Mater. Electron..

[19] Nazeeruddin MK, Kay A, Rodicio I, Humphry-Baker R, Müller E, Liska P, Vlachopoulos N, Grätzel M (1993). Conversion of light to electricity by cis-X2bis (2, 2'-bipyridyl-4, 4′-dicarboxylate) ruthenium(II) charge-transfer sensitizers (X= Cl-, Br-, I-, CN-, and SCN-) on nanocrystalline titanium dioxide electrodes. J. Am. Chem. Soc..

[20] Nazeeruddin MK, Pechy P, Renouard T, Zakeeruddin SM, Humphry-Baker R, Comte P, Liska P, Cevey L, Costa E, Shklover V, Spiccia L (2001). Engineering of efficient panchromatic sensitizers for nanocrystalline TiO_2_-based solar cells. J. Am. Chem. Soc..

[21] Ansari MIH, Qurashi A, Nazeeruddin MK (2018). Frontiers, opportunities, and challenges in perovskite solar cells: A critical review. J. Photochem. Photobiol. C Photochem. Rev..

[22] Mathew S, Yella A, Gao P, Humphry-Baker R, Curchod BF, Ashari-Astani N, Tavernelli I, Rothlisberger U, Nazeeruddin M, Grätzel M (2014). Dye-sensitized solar cells with 13% efficiency achieved through the molecular engineering of porphyrin sensitizers. Nat. Chem..

[23] Giribabu L, Kanaparthi RK, Velkannan V (2012). Molecular engineering of sensitizers for dye-sensitized solar cell applications. Chem. Rec..

[24] Abdelhamed FH, Ismail MA, Abdel-Latif E, Abdel-Shafi AA, Elmorsy MR (2022). Design and synthesis of novel bichalcophene derivatives with double anchoring groups for dye-sensitized solar cell applications: Sensitization and co-sensitization with N-719. J. Mater. Sci. Mater. Electron..

[25] Kharkwal D, Sharma N, Gupta SK, Negi CMS (2021). Enhanced performance of dye-sensitized solar cells by co-sensitization of metal-complex and organic dye. Sol. Energy.

[26] Koteshwar D, Prasanthkumar S, Singh SP, Chowdhury TH, Bedja I, Islam A, Giribabu L (2022). Effects of methoxy group (s) on D–π–A porphyrin based DSSCs: Efficiency enhanced by co-sensitization. Mater. Chem. Front..

[27] Alnakeeb A, Fadda AA, Ismail MA, Elmorsy MR (2022). Efficient co-sensitization of novel trimethoxybenzene-based dyes with N-719 for highly efficient dye-sensitized solar cells. Opt. Mater..

[28] Devadiga D, Selvakumar M, Devadiga D, Ahipa TN, Shetty P, Paramasivam S, Kumar SS (2022). The improved performance of dye-sensitized solar cells using co-sensitization and polymer gel electrolyte. Int. J. Energy Res..

[29] Ismail MA (2006). An efficient synthesis of 5'-(4-cyanophenyl)-2,2'-bifuran-5-carbonitrile and analogues. J. Chem. Res..

[30] Yokooji A, Satoh T, Miura M, Nomura M (2004). Synthesis of 5, 5′-diarylated 2,2′-bithiophenes via palladium-catalyzed arylation reactions. Tetrahedron.

[31] Ismail MA, Shaban MM, Abdel-Latif E, Abdelhamed FH, Migahed MA, El-Haddad MN, Abousalem AS (2022). Novel cationic aryl bithiophene/terthiophene derivatives as corrosion inhibitors by chemical, electrochemical and surface investigations. Sci. Rep..

[32] Ismail MA, Abdel-Rhman MH, Abdelwahab GA, Hamama WS (2020). Synthesis and spectroscopic studies of methoxysubstituted phenylthienylnicotinamidines. Synth. Commun..

[33] Yang J, Ganesan P, Teuscher J, Moehl T, Kim YJ, Yi C, Comte P, Pei K, Holcombe TW, Nazeeruddin MK, Hua J, Zakeeruddin SM, Tian H, Grätzel M (2014). Influence of the donor size in D–π–A organic dyes for dye-sensitized solar cells. J. Am. Chem. Soc..

[34] Blackbourn RL, Johnson CS, Hupp JT (1991). Surface intervalence enhanced Raman scattering from ferrocyanide on colloidal titanium dioxide. A mode-by-mode description of the Franck–Condon barrier to interfacial charge transfer. J. Am. Chem. Soc..

[35] Yang M, Thompson DW, Meyer G (2002). Charge-transfer studies of iron cyano compounds bound to nanocrystalline TiO_2_ surfaces. J. Inorg. Chem..

[36] Khoudiakov M, Parise AR, Brunschwig BS (2003). Interfacial electron transfer in Fe^II^(CN)_6_^4^^−^–sensitized TiO_2_ nanoparticles: A study of direct charge injection by electroabsorption spectroscopy. J. Am. Chem. Soc..

[37] Lu H, Prieskorn JN, Hupp JT (1993). Fast interfacial electron transfer: Evidence for inverted region kinetic behaviour. J. Am. Chem. Soc..

[38] Weng Y-X, Wang Y-Q, Asbury JB, Ghosh HN, Lian T (2000). Back electron transfer from TiO_2_ nanoparticles to Fe^III^(CN)_6_^3^^−^: Origin of non-single-exponential and particle size independent dynamics. J. Phys. Chem. B..

[39] Ramakrishna G, Ghosh HN (2001). Emission from the charge transfer state of xanthene dye-sensitized TiO_2_ nanoparticles: A new approach to determining back electron transfer rate and verifying the marcus inverted regime. J. Phys. Chem. B..

[40] Walters KA, Gaal DA, Hupp JT (2002). Interfacial charge transfer and colloidal semiconductor dye-sensitization: Mechanism assessment via Stark emission spectroscopy. J. Phys. Chem. B..

[41] Reddy PY, Giribabu L, Lyness C, Snaith HJ, Vijaykumar C, Chandrasekharam M, Lakshmikantam M, Yum J-H, Kalyanasundaram K, Grätzel M, Nazeeruddin MK (2007). Angew. Chem. Int. Ed..

[42] Basham JI, Mor GK, Grimes CA (2010). ACSNANO.

[43] Luo J, Wan Z, Jia C, Wang Y, Wu X, Yao X (2016). Co-sensitization of dithiafulvenyl-phenothiazine based organic dyes with N719 for efficient dye-sensitized solar cells. Electrochim. Acta.

[44] Huang Z-S, Feng H-L, Zang X-F, Iqbal Z, Zeng H, Kuang D-B, Wang L, Meier H, Cao D (2014). Dithienopyrrolobenzothiadiazole-based organic dyes for efficient dye-sensitized solar cells. J. Mater. Chem. A..

[45] Tian H, Yang X, Cong J, Chen R, Teng C, Liu J, Hao Y, Wang L, Sun L (2010). Effect of different electron donating groups on the performance of dye-sensitized solar cells. Dyes Pigm..

[46] Zhao J, Yang X, Cheng M, Li S, Sun L (2013). Molecular design and performance of hydroxylpyridium sensitizers for dye-sensitized solar cells. ACS Appl. Mater. Interfaces.

[47] Frisch, M. J., Trucks, G. W., Schlegel, H. B., Scuseria, G. E., Robb, M. A., Cheeseman, J. R., Scalmani, G., Barone, V., Mennucci, B., Petersson, G. A., Nakatsuji, H., Caricato, M., Li, X., Hratchian, H. P., Izmaylov, A. F., Bloino, J., Zheng, G., Sonnenberg, J. L., Hada, M., Ehara, M., Toyota, K., Fukuda, R., Hasegawa, J., Ishida, M., Nakajima, T., Honda, Y., Kitao, O., Nakai, H., Vreven, T., Montgomery Jr., J. A., Peralta, J. E., Ogliaro, F., Bearpark, M., Heyd, J. J., Brothers, E., Kudin, K. N., Staroverov, V. N., Kobayashi, R., Normand, J., Raghavachari, K., Rendell, A., Burant, J. C., Iyengar, S. S., Tomasi, J., Cossi, M., Rega, N., Millam, J. M., Klene, M., Knox, J. E., Cross, J. B., Bakken, V., Adamo, C., Jaramillo, J., Gomperts, R., Stratmann, R. E., Yazyev, O., Austin, A. J., Cammi, R., Pomelli, C., Ochterski, J. W., Martin, R. L., Morokuma, K., Zakrzewski, V. G., Voth, G. A., Salvador, P., Dannenberg, J. J., Dapprich, S., Daniels, A. D., Farkas, O., Foresman, J. B., Ortiz, J. V., Cioslowski, J. & Fox, D. J. (Gaussian 09 Gaussian, Inc., 2010).

[48] Nielsen, A. B. & Holder, A. J. *Gauss View 5.0, User’s Reference* (GAUSSIAN Inc., 2009)

[49] Lee CT, Yang WT, Parr RG (1988). Development of the Colle–Salvetti correlation-energy formula into a functional of the electron density. Phys. Rev. B.

[50] Arbelo-Lópezlópez HD, Rodriguez-Mackenzie AD, Roman-Morales EM, Wymore T, Lópezlópez-Garriga J, Mayagü Ez Campus R, Ez M, Rico P (2018). Charge transfer and π to π* transitions in the visible spectra of sulfheme met isomeric structures. J. Phys. Chem. B.

[51] Murray JS, Sen K (1996). Molecular Electrostatic Potentials: Concepts and Applications.

[52] Shakila G, Saleem H, Sundaraganesan N (2017). FT-IR, FT-Raman, NMR and U–V Spectral investigation: Computation of vibrational frequency, chemical shifts and electronic structure calculations of 1-bromo-4-nitrobenzene. World Sci. News.

[53] Raftani M, Abram T, Bennani MN, Bouachrine M (2020). Theoretical study of new conjugated compounds with a low bandgap for bulk heterojunction solar cells: DFT and TD-DFT study. Results Chem..

[54] Neale NR, Kopidakis N, De Lagemaat JV, Gratzel M, Frank AJ (2005). Effect of a coadsorbent on the performance of dye-sensitized TiO_2_ solar cells: shielding versus band-edge movement. J. Phys. Chem. B.

[55] Ito S, Miura H, Uchida S, Takata M, Sumioka K, Liska P, Comte P, Pechy P, Gratzel M (2008). High-conversion-efficiency organic dye-sensitized solar cells with a novel indoline dye. Chem Commun..

[56] El-Shafei A, Hussain M, Atiq A, Islam A, Han L (2012). A novel carbazole-based dye outperformed the benchmark dye N719 for high efficiency dye-sensitized solar cells (DSSCs). J. Mater. Chem..

[57] Yan R, Qian X, Jiang Y, He Y, Hang Y, Hou L (2017). Ethynylene-linked planar rigid organic dyes based on indeno [1, 2-b] indole for efficient dye-sensitized solar cells. Dyes Pigm..

[58] Oskam G, Bergeron BV, Meyer GJ, Searson PC (2001). Pseudohalogens for dye-sensitized TiO_2_ photoelectrochemical cells. J. Phys. Chem. B.

[59] Hua Y, Jin B, Wang H, Zhu X, Wu W, Cheung MS, Lin Z, Wong WY, Wong WK (2013). Bulky dendritic triarylamine-based organic dyes for efficient co-adsorbent-free dye-sensitized solar cells. J. Power Sources.

[60] Qu P, Meyer GJ (2001). Proton-controlled electron injection from molecular excited states to the empty states in nanocrystalline TiO_2_. Langmuir.

