# Supplementary material for: Design, synthesis, and performance evaluation of TiO2-dye sensitized solar cells using 2,2′-bithiophene-based co-sensitizers
Source: Sci Rep. 2023 Aug 24;13:13825. doi: 10.1038/s41598-023-40830-1 (PMC10449855; doi:10.1038/s41598-023-40830-1)
Supplement: Supplementary file 1 — Supplementary Information. [file 41598_2023_40830_MOESM1_ESM.docx]

**Design, Synthesis, and Performance Evaluation of TiO_2_-Dye Sensitized Solar Cells Using 2,2'-Bithiophene-Based Co-Sensitizers.**

Mohamed R. Elmorsy^a,^*, Fatma H. Abdelhamed^a^, Safa A. Badawy^a^, Ehab Abdel-Latif^a^, Ayman A. Abdel-Shafi^b^, Mohamed A. Ismail^a^

*^a^Department of Chemistry, Faculty of Science, Mansoura University, El-Gomhoria Street, 35516 Mansoura, Egypt.*

*^b^Department of Chemistry, Faculty of Science, Ain Shams University, Abbassia, 11566,Cairo, Egypt.*

*^*^ Corresponding author: E-mail:* m.r.elmorsy@gmail.com

**1. General remarks, Chemicals and equipment's**

Melting points were measured in degree centigrade on Gallenkamp apparatus and are uncorrected. The reaction mixture was monitored by using thin-layer chromatography (TLC) which was made by using silica gel 60 F_254_ precoated aluminum sheets, visualized by ultraviolet (UV) light. The ultraviolet-visible (UV-Vis) absorption spectra were measured on a Schimadzu 1900 spectrophotometer in the region of 200–600 nm. Fluorescence measurements were obtained using RF-6000 Spectrofluorophotometer. The infrared spectra (KBr) were explored on Thermo Scientific Nicolet iS10 FTIR spectrometer. ^1^H-NMR (500 MHz) spectra were measured on JEOL’s spectrometer using DMSO-*d_6_* as a solvent and self-internal standard. A Schimadzu Qp-2010 Plus (GC-MS) spectrometer was used for recording mass spectra of the newly synthesized compounds. Elemental analyses were performed on Perkin-Elmer 2400 analyser. All chemicals and solvents were purchased from Aldrich Chemical Co., Fisher Scientific. All solvents were reagent grade.

**General methodology for preparation of 2,2’-bithiophene carbonitriles 3a-c**

To a stirred solution of 4-(5'-bromo-[2,2'-bithiophen]-5-yl)benzonitrile **1** (692 mg, 2 mmol), Pd(PPh_3_)_4_ (120 mg), anhy. K_2_CO_3_ (3 g) in 20 mL dioxane was added the proper phenylboronic acid (2.4 mmol). The reaction mixture was refluxed for ~12 hrs, monitored by TLC and then 5 mL of conc. NH_4_OH was added and the reaction mixture was extracted with CH_2_Cl_2_ (250 mL, x3). The organic layer was evaporated, and the resultant precipitate was recrystallized from DMF/EtOAc affording bithienylbenzonitrile derivatives **3a-c**.

***4-{5'-(4-Methoxyphenyl)-[2,2'-bithiophen]-5-yl}benzonitrile (3a).***

**3a** was obtained in 75% yield as a yellowish-orange solid, m.p. = 189-190 ^o^C, lit. [1] m.p. =187-188 ^o^C. R_f_ = 0.57, petroleum ether-EtOAc (8:2). IR (KBr) ν/cm^-1^; 3060, 2928 (CH), 2228 (CN), 1603, 1528, 1495 (C=C). ^1^H-NMR (DMSO-*d*_6_); δ ppm 3.78 (s, 3H), 6.99 (d, J = 8.0 Hz, 2H), 7.38-7.41 (m, 3H), 7.61 (d, J = 8.0 Hz, 2H), 7.74 (d, J = 4.0 Hz, 1H), 7.86 (s, 4H). MS (EI) m/e (rel.int.); 373 (M^+^, 100), 358 (M^+^-CH_3_, 57), 330 (20). Anal. Calc. for C_22_H_15_NOS_2_ (373.49): C, 70.75; H, 4.05; N, 3.75. Found: C, 70.63; H, 4.18; N, 3.56.

***4-{5'-(3,4-Dimethoxyphenyl)-[2,2'-bithiophen]-5-yl}benzonitrile (3b).***

**3b** was obtained in 77% yield as a yellowish-orange solid, m.p. 199-200 ^o^C. R_f_ = 0.68, petroleum ether-EtOAc (8:2). IR (KBr) ν/cm^-1^; 2960 (CH), 2221 (CN), 1600, 1524, 1460 (C=C). ^1^H-NMR (DMSO-*d*_6_); δ ppm 3.78 (s, 3H), 3.83 (s, 3H), 6.99 (d, J = 8.5 Hz, 1H), 7.20 (dd, J = 8.5, 2.5 Hz, 1H), 7.22 (d, J = 2.5 Hz, 1H), 7.40 (d, J = 3.5 Hz, 1H), 7.41 (d, J = 3.5 Hz, 1H), 7.46 (d, J = 4.0 Hz, 1H), 7.74 (d, J = 4.0 Hz, 1H), 7.86 (s, 4H). MS (EI) m/e (rel.int.); 403 (M^+^, 100), 388 (M^+^- CH_3_, 22). Anal. Calc. for C_23_H_17_NO_2_S_2_ (403.51): C, 68.46; H, 4.25; N, 3.47. Found: C, 68.21; H, 4.34; N, 3.56.

***4-{5'-(3,5-Dimethoxyphenyl)-[2,2'-bithiophen]-5-yl}benzonitrile (3c).***

**3c** was obtained in 83% yield as a yellowish-orange solid, m.p. 206-208^o^C. R_f_ = 0.63, petroleum ether-EtOAc (8:2). IR (KBr) ν/cm^-1^; 2924 (CH), 2220 (CN), 1585, 1453 (C=C). ^1^H-NMR (DMSO-*d*_6_); δ ppm 3.79 (s, 6H), 6.48-6.49 (m, 1H), 6.81 (d, J = 2.0 Hz, 2H), 7.43 (d, J = 3.5 Hz, 1H), 7.46 (d, J = 4.0 Hz, 1H), 7.58 (d, J = 3.5 Hz, 1H) , 7.76 (d, J = 4.0 Hz, 1H), 7.87 (s, 4H). MS (EI) m/e (rel.int.); 403 (M^+^, 100), 360 (10), 317(20). Anal. Calc. for C_23_H_17_NO_2_S_2_ (403.51): C, 68.46; H, 4.25; N, 3.47. Found: C, 68.23; H, 4.32; N, 3.36.

**Preparation of 2,2’-bithiophene fluorobenzonitriles 5a-c** was made adopting the same Suzuki coupling condition used for preparation of bithienylbenzonitriles **3a-c** using 4-(5'-bromo-[2,2'-bithiophen]-5-yl)-2-fluorobenzonitrile **4**, instead of bromo derivative **1**.

***2-Fluoro-4-{5'-(4-methoxyphenyl)-[2,2'-bithiophen]-5-yl}benzonitrile (5a).***

**5a** was obtained in 70% yield as a golden-yellow solid, m.p. 189-190^o^C. R_f_ = 0.53, petroleum ether-EtOAc (8:2). IR (KBr) ν/cm^-1^; 2925 (CH), 2228 (CN), 1611, 1528, 1424 (C=C). ^1^H-NMR (DMSO-*d*_6_); δ ppm 3.78 (s, 3H), 6.98 (d, J = 8.5 Hz, 2H), 7.39-7.42 (m, 2H), 7.43 (d, J = 3.5 Hz, 1H), 7.61 (d, J = 8.5 Hz, 2H), 7.67 (dd, J = 8.0, 1.5 Hz, 1H), 7.82 (d, J = 3.5 Hz, 1H), 7.87-7.94 (m, 2H). MS (EI) m/e (rel.int.); 392 (M^+^+1, 28), 391 (M^+^, 100), 376 (M^+^-CH_3_, 52). Anal. Calc. for C_22_H_14_FNOS_2_ (391.48): C, 67.50; H, 3.60; N, 3.58. Found: C, 67.21; H, 3.68; N, 3.49.

***4-{5'-(3,4-Dimethoxyphenyl)-[2,2'-bithiophen]-5-yl}-2-fluorobenzonitrile (5b).***

**5b** was obtained in 67% yield as a yellowish-brown solid, m.p. 150-152^o^C. R_f_ = 0.64, petroleum ether-EtOAc (8:2). IR (KBr) ν/cm^-1^; 2928, 2839 (CH), 2228 (CN), 1613, 1528, 1438 (C=C). ^1^H-NMR (DMSO-*d*_6_); δ ppm 3.78 (s, 3H), 3.83 (s, 3H), 6.99 (d, J = 8.0 Hz, 1H), 7.22 (dd, J = 8.0, 1.5 Hz, 1H), 7.25 (d, J = 1.5 Hz, 1H), 7.38-7.41 (m, 1H), 7.45 (d, J = 4.0 Hz, 1H), 7.55 (d, J = 3.5 Hz, 1H), 7.65 (d, J = 8.0 Hz, 1H), 7.83 (d, J = 4.0 Hz, 1H), 7.85-7.93 (m, 2H). MS (EI) m/e (rel.int.); 421 (M^+^, 100), 406 (M^+^- CH_3_, 22). Anal. Calc. for C_23_H_16_FNO_2_S_2_ (421.50): C, 65.54; H, 3.83; N, 3.32. Found: C, 65.33; H, 3.89; N, 3.24.

***4-{5'-(3,5-Dimethoxyphenyl)-[2,2'-bithiophen]-5-yl}-2-fluorobenzonitrile (5c).***

**5c** was obtained in 71% yield as a yellowish-orange solid, m.p. 152-154^o^C. R_f_ = 0.61, petroleum ether-EtOAc (8:2). IR (KBr) ν/cm^-1^; 2926, 2851 (CH, stretch), 2229 (CN), 1615, 1590, 1420 (C=C). ^1^H-NMR (DMSO-*d*_6_); δ ppm 3.79 (s, 6H), 6.47-6.48 ppm (m, 1H), 6.79 (d, J =1.5 Hz, 2H), 7.43(d, J = 4.0 Hz, 1H), 7.46 (d, J = 4.0 Hz, 1H), 7.57(d, J = 4.0 Hz, 1H), 7.66 (dd, J = 8.0, 1.5 Hz, 1H), 7.82 (d, J = 4.0 Hz, 1H), 7.86-7.94 (m, 2H). MS (EI) m/e (rel.int.); 421 (M^+^, 52), 69 (100). Anal. Calc. for C_23_H_16_FNO_2_S_2_ (421.50): C, 65.54; H, 3.83; N, 3.32. Found: C, 65.26; H, 3.90; N, 3.22.

**3. Spectral analysis:**

| **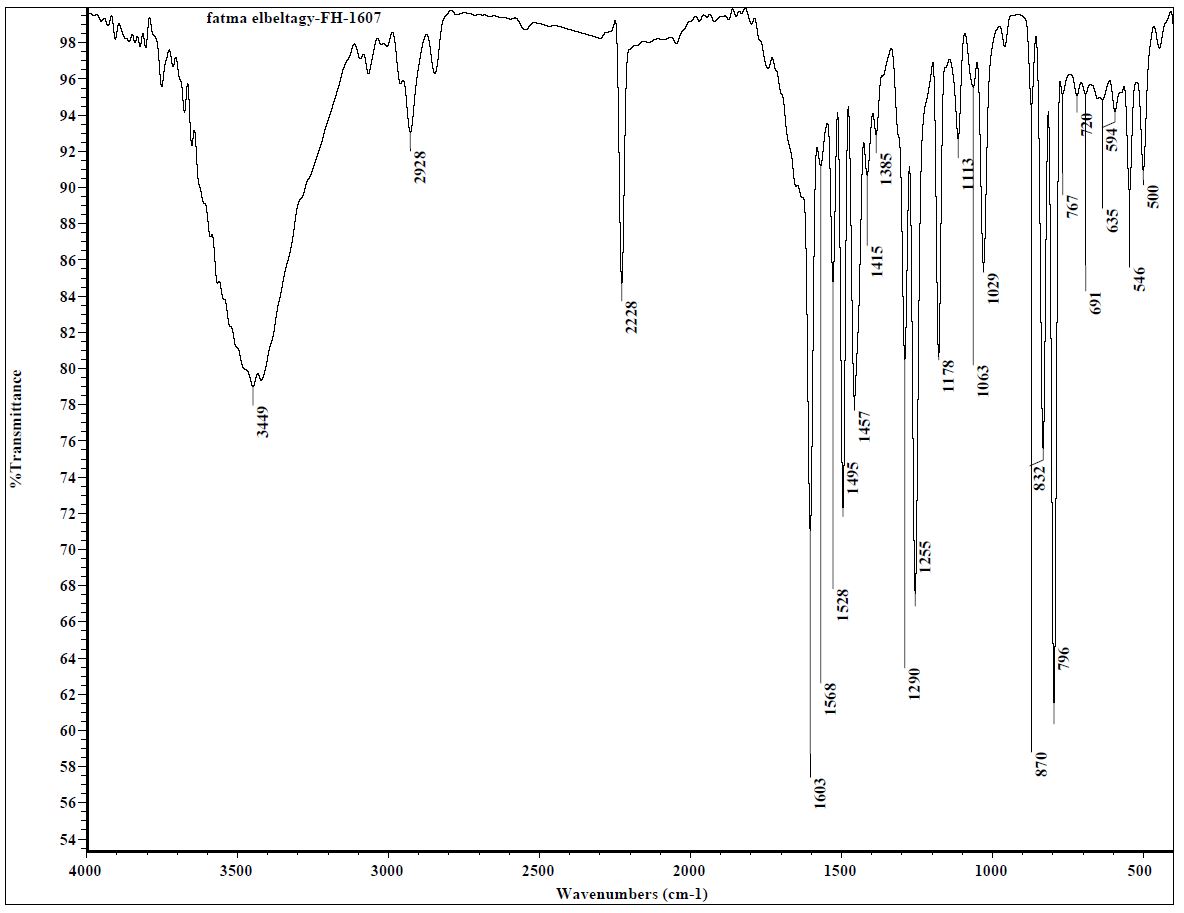**    **Figure (1): IR spectrum of compound 3a** |
| --- |

| 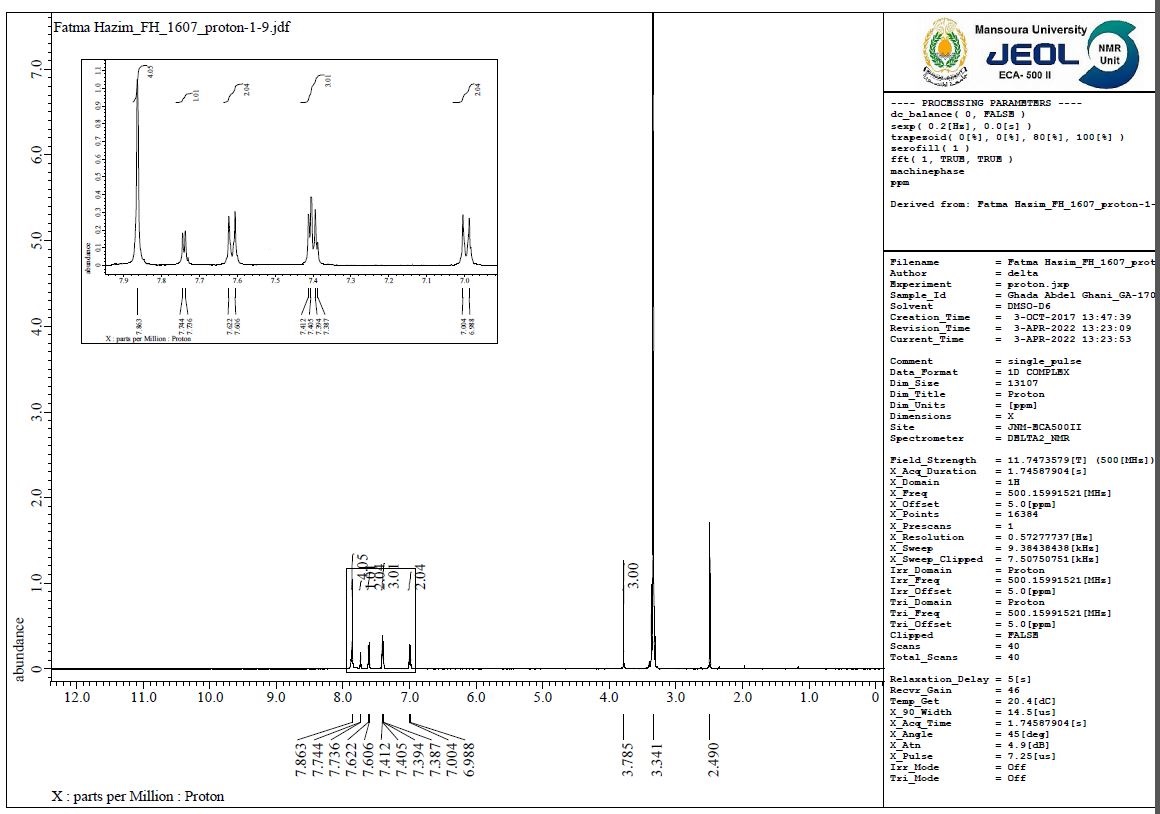    **Figure (2): ^1^H-NMR spectrum of compound 3a** |
| --- |

| ****  .  **Figure (3): Mass spectrum of compound 3a** |
| --- |

| **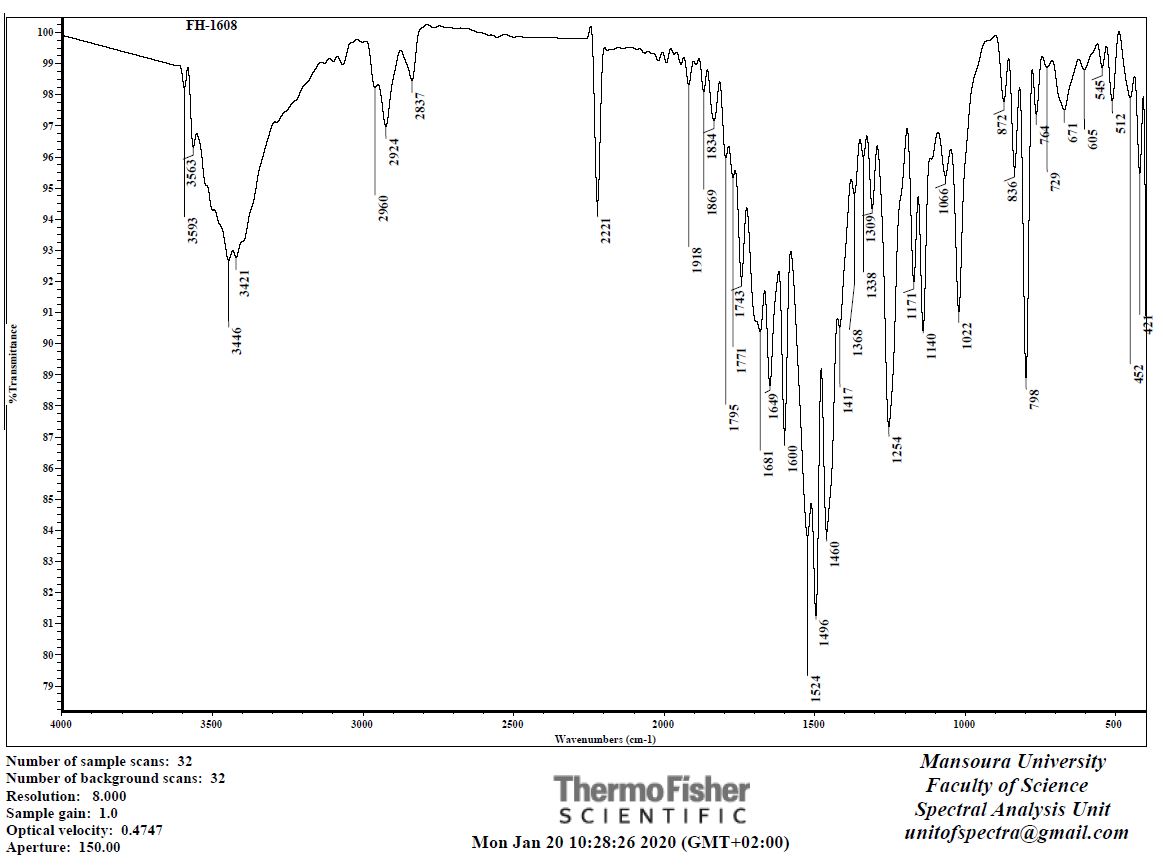**    **Figure (4): IR spectrum of compound 3b** |
| --- |

| **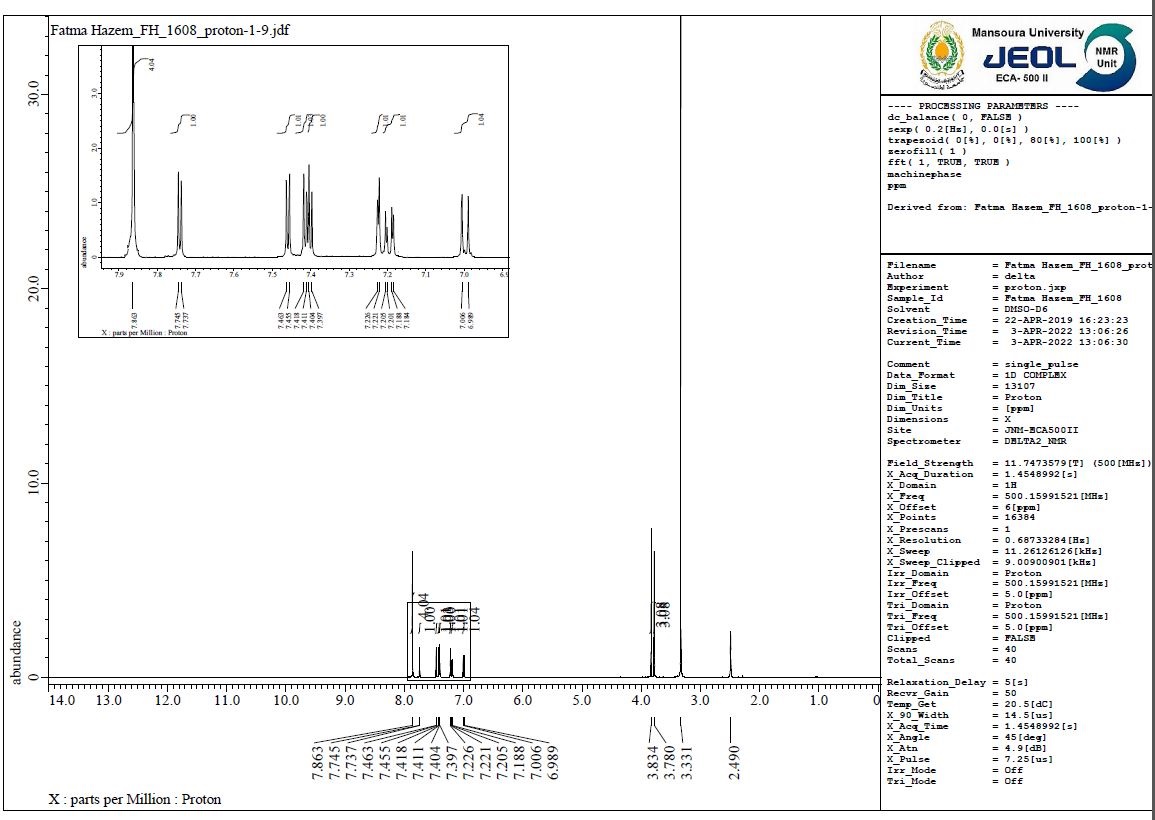**    **Figure (5): ^1^H-NMR spectrum of compound 3b** |
| --- |

| ****    **Figure (6): Mass spectrum of compound 3b** |
| --- |

| **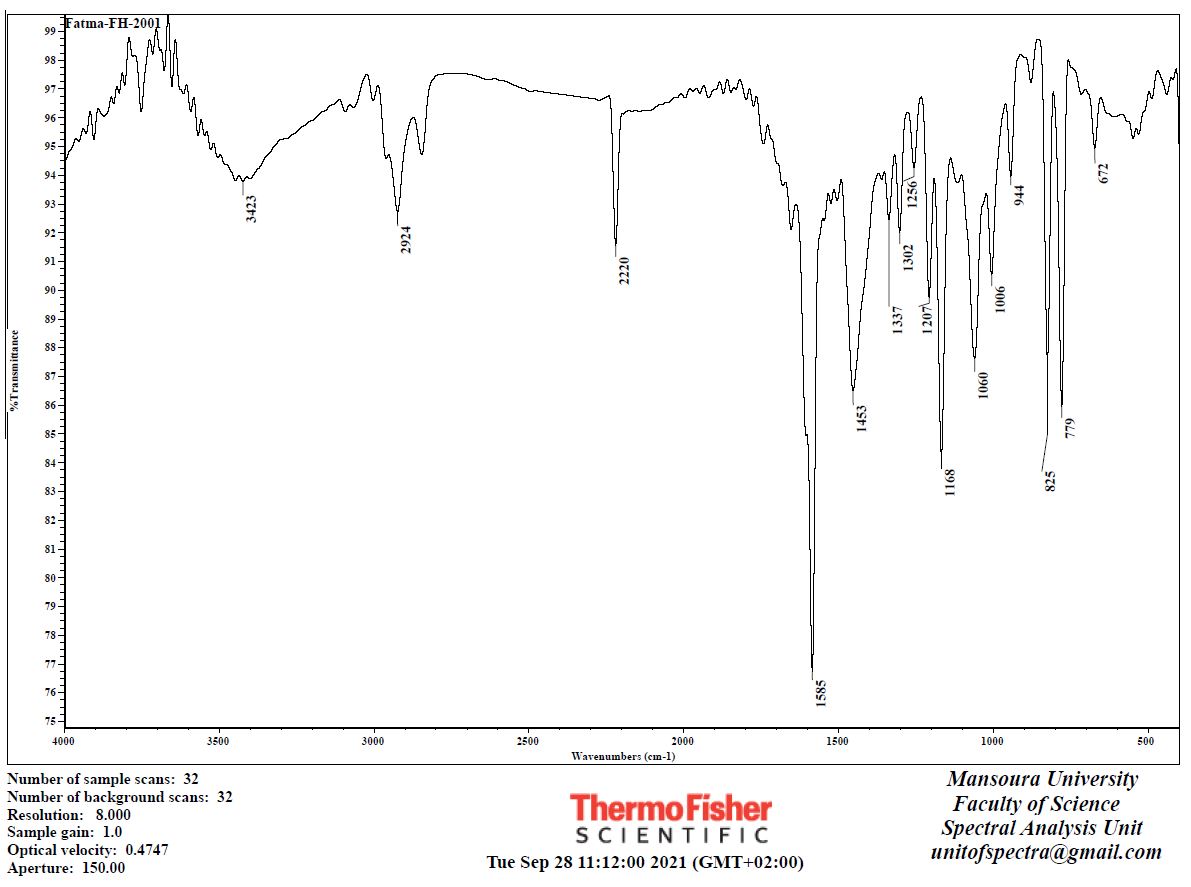**    **Figure (7): IR spectrum of compound 3c** |
| --- |

| 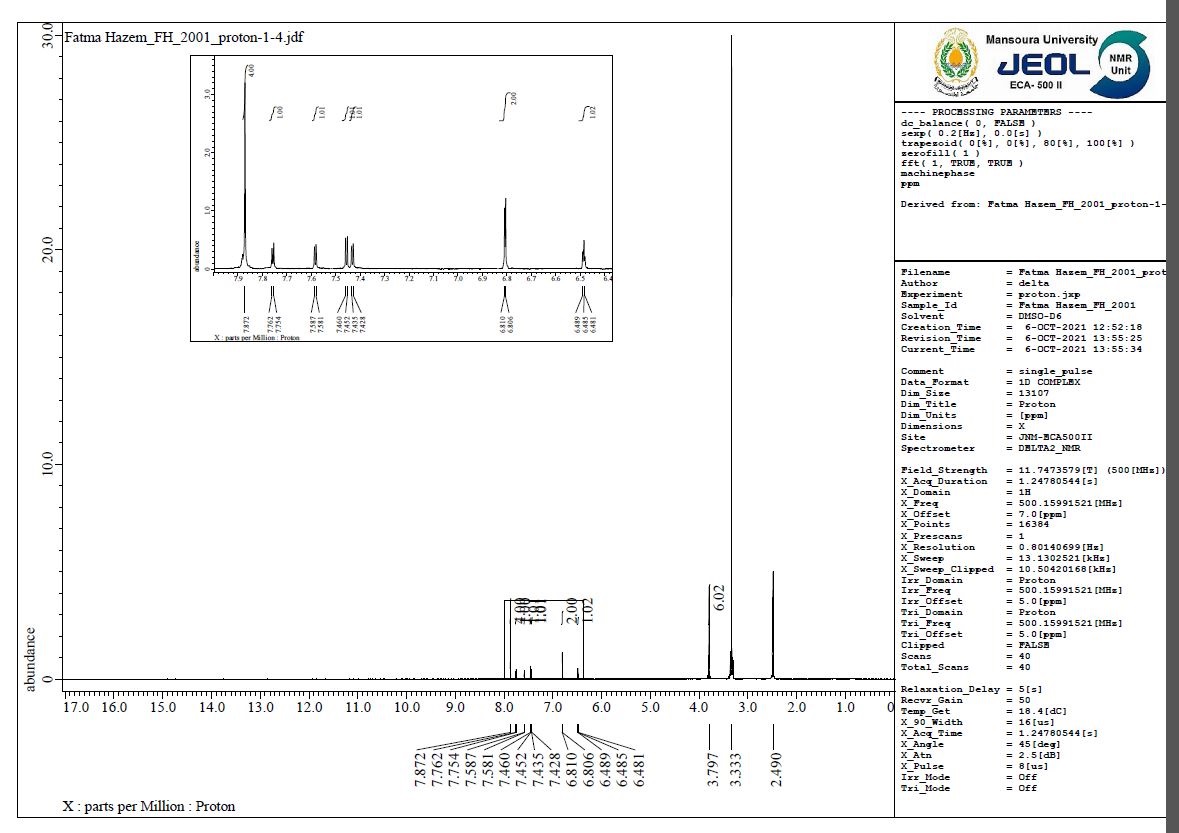    **Figure (8): ^1^H-NMR spectrum of compound 3c** |
| --- |

| 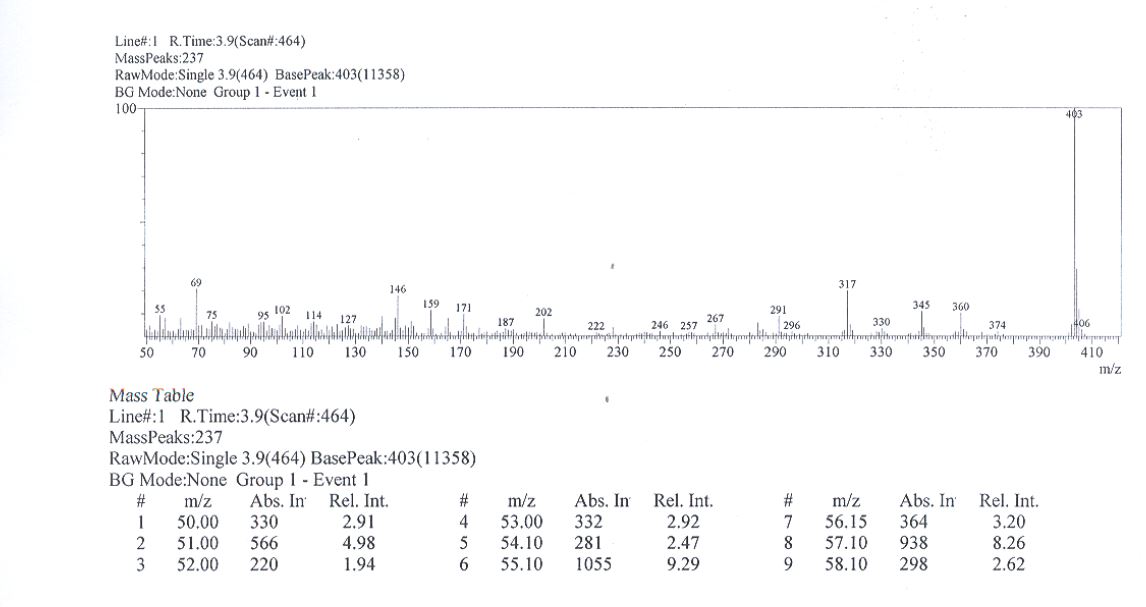    **Figure (9): Mass spectrum of compound 3c** |
| --- |

| 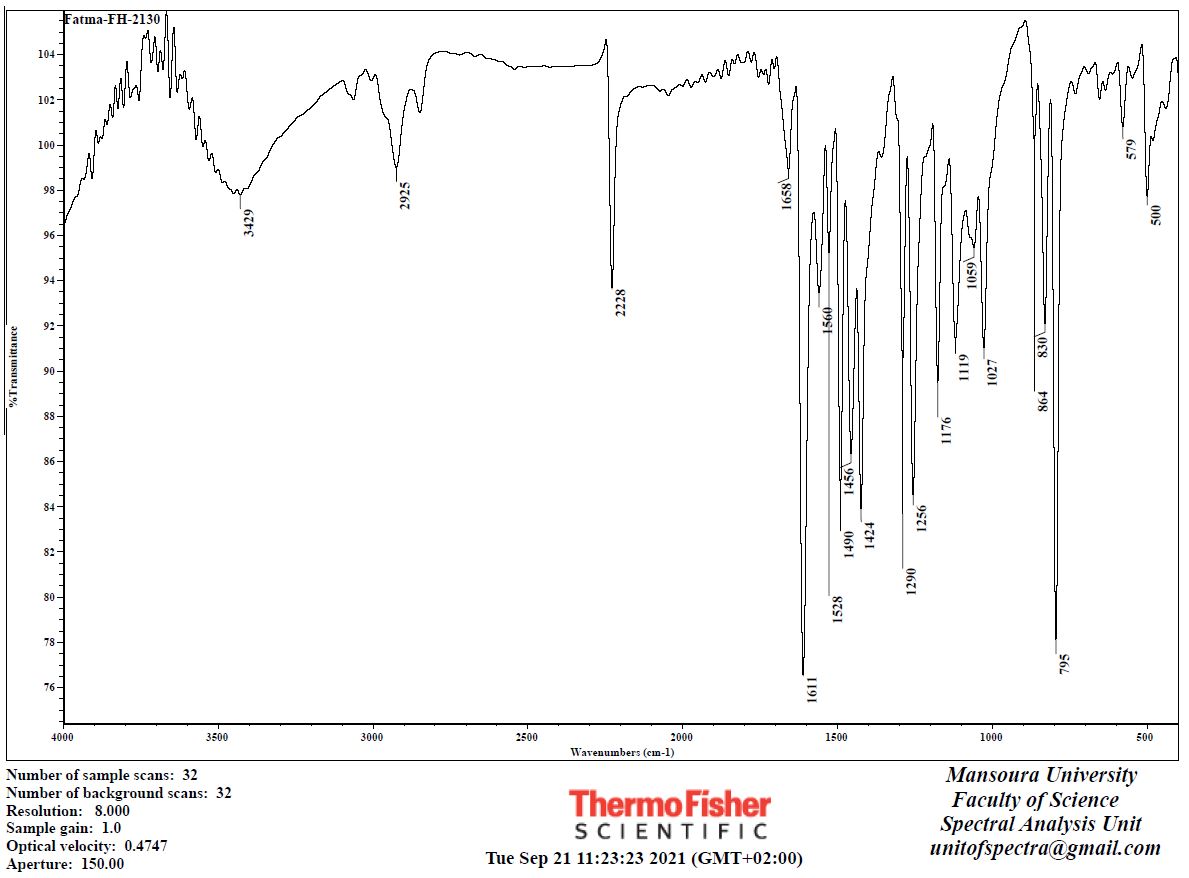    **Figure (10): IR spectrum of compound 5a** |
| --- |

| **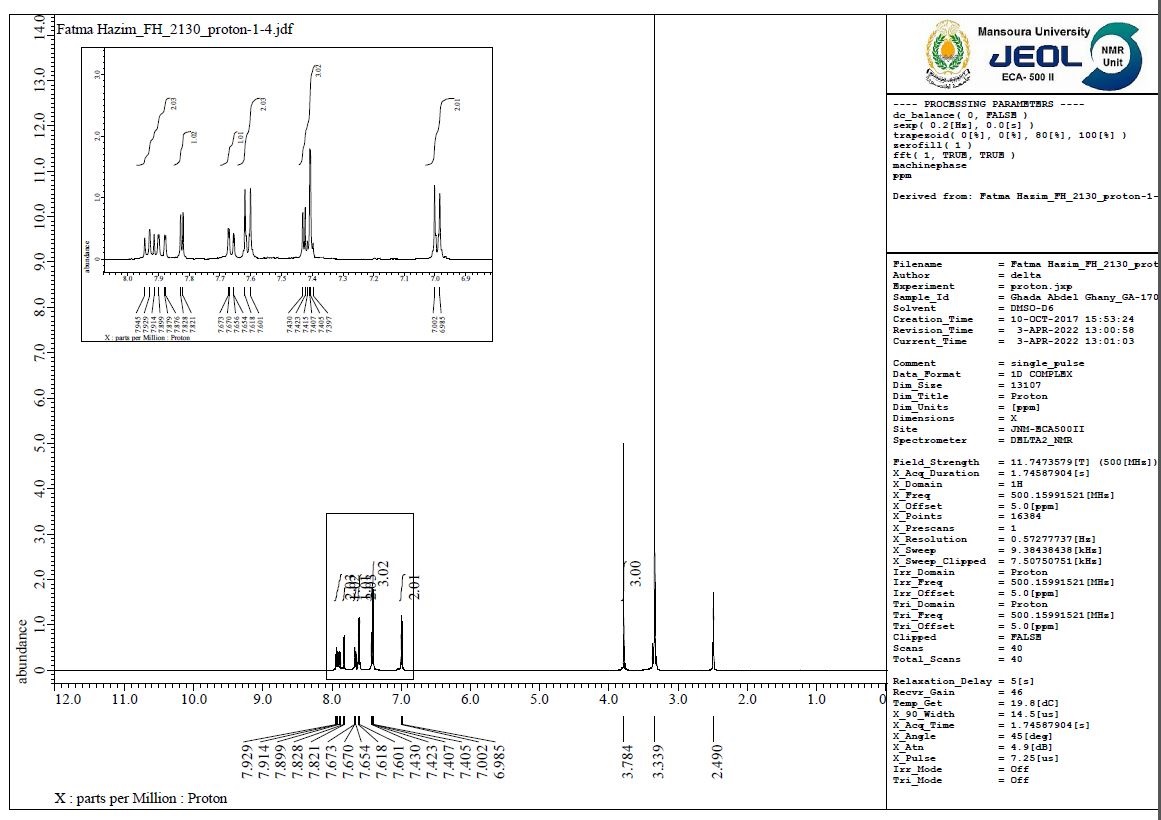**    **Figure (11): ^1^H-NMR spectrum of compound 5a** |
| --- |

| **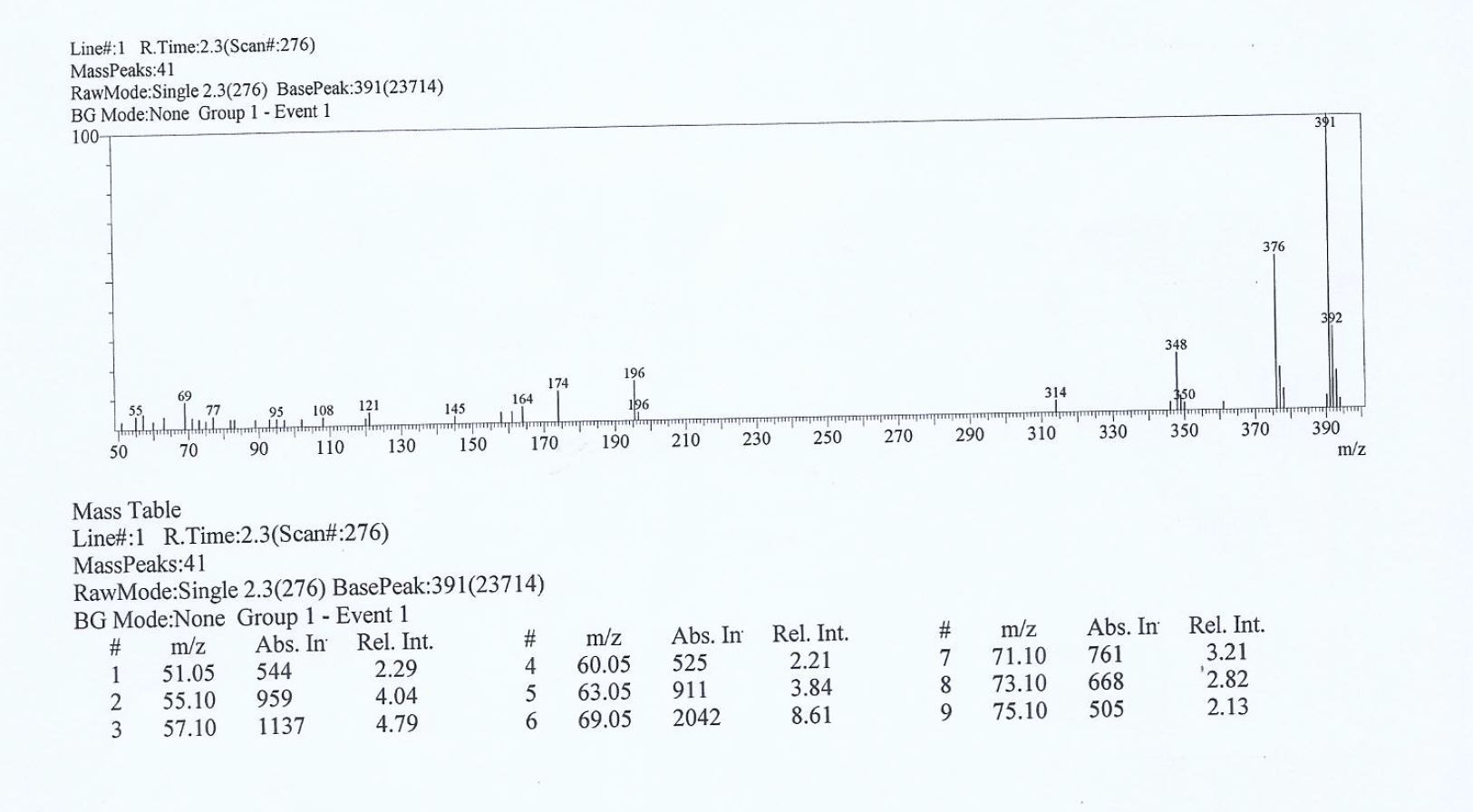**    **Figure (12): Mass spectrum of compound 5a** |
| --- |

| **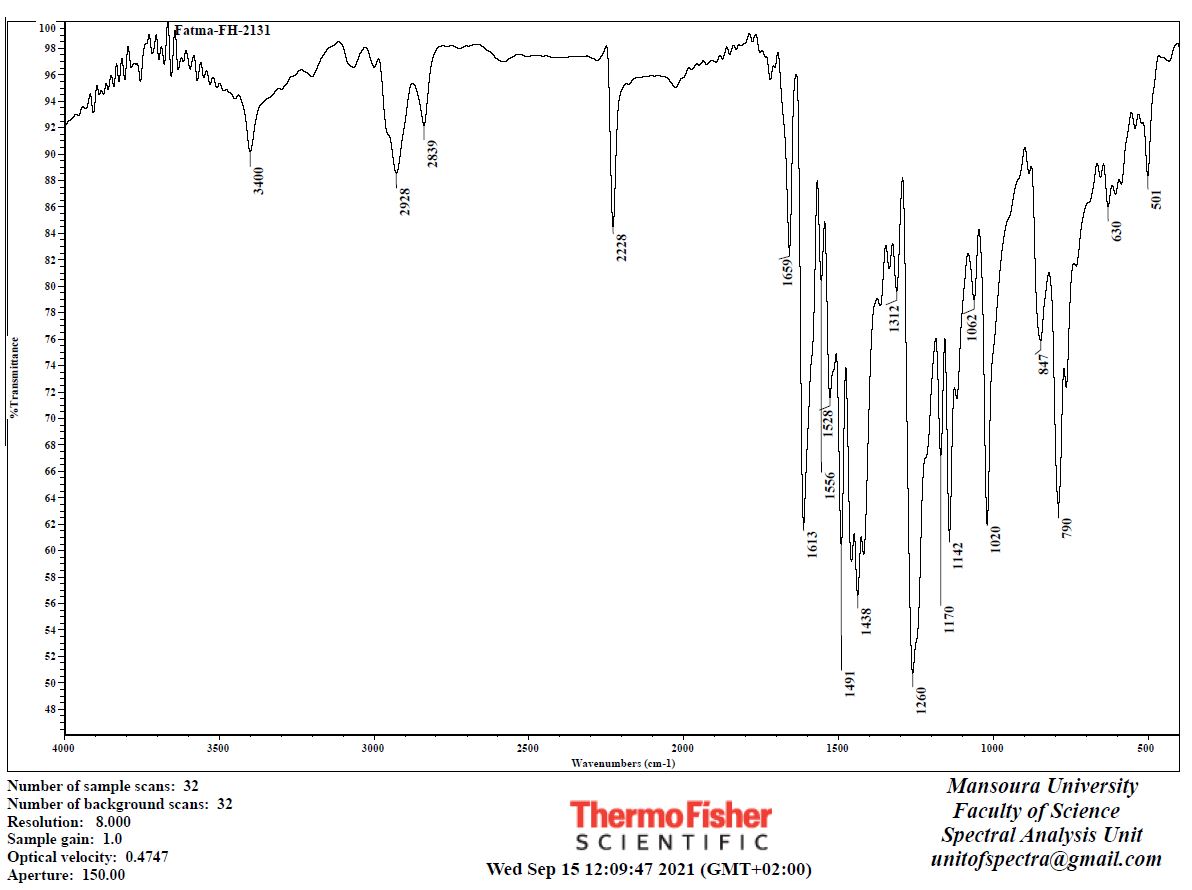**    **Figure (13): IR spectrum of compound 5b** |
| --- |

| **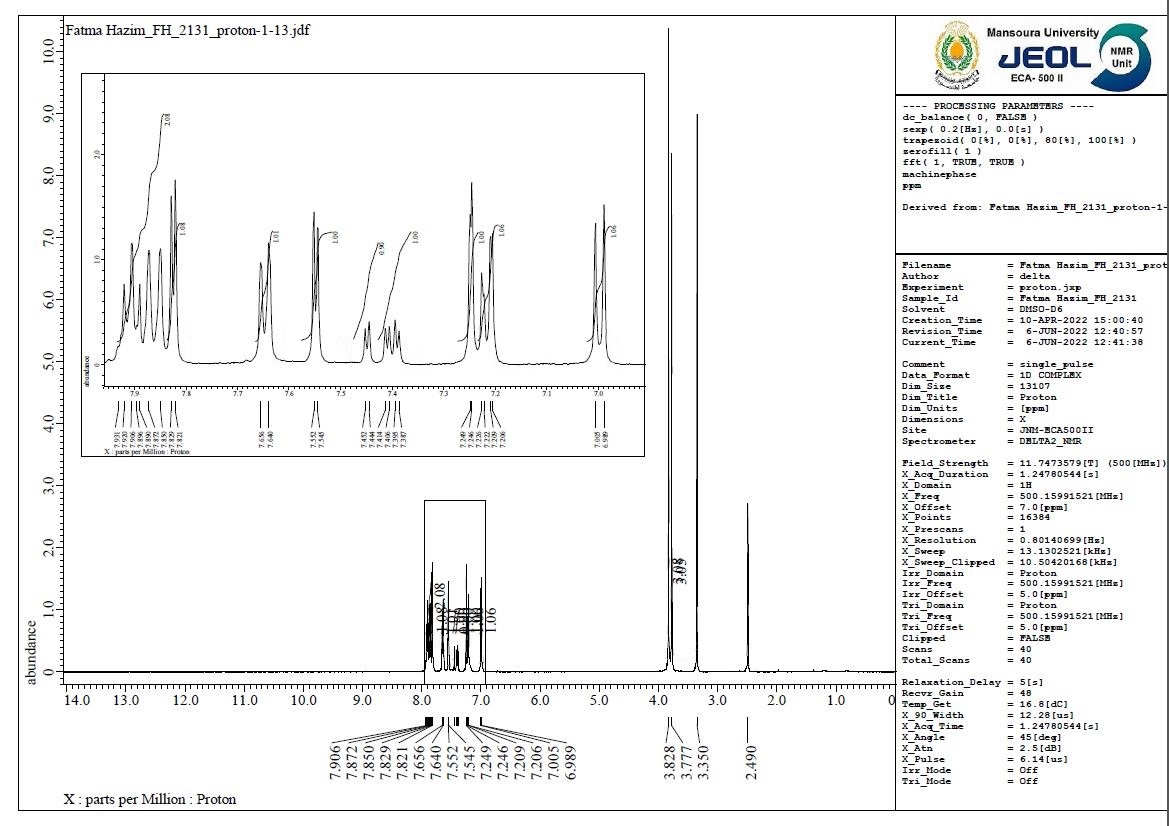**    **Figure (14): ^1^H-NMR spectrum of compound 5b** |
| --- |

| **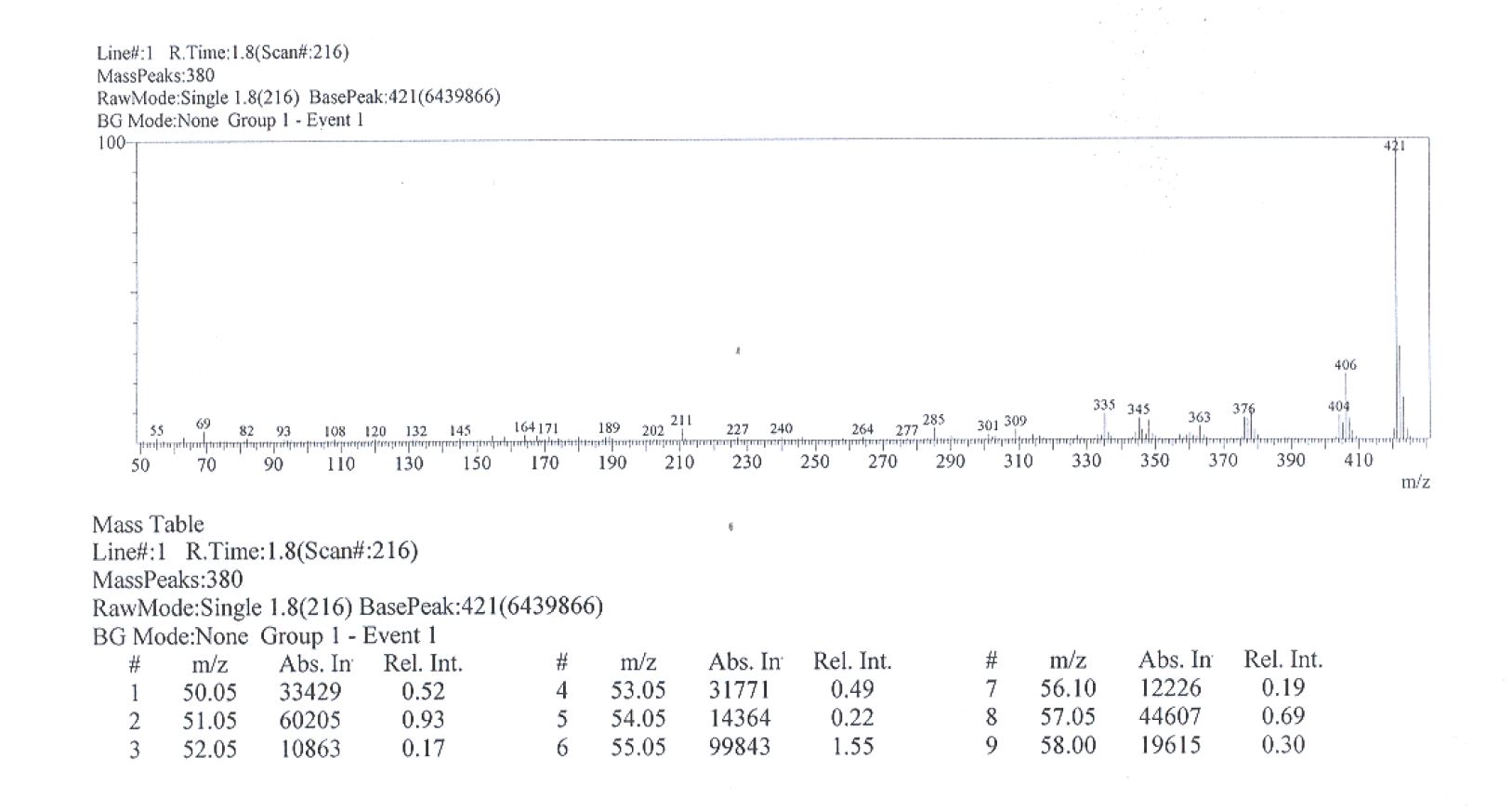**    **Figure (15): Mass spectrum of compound 5b** |
| --- |

| **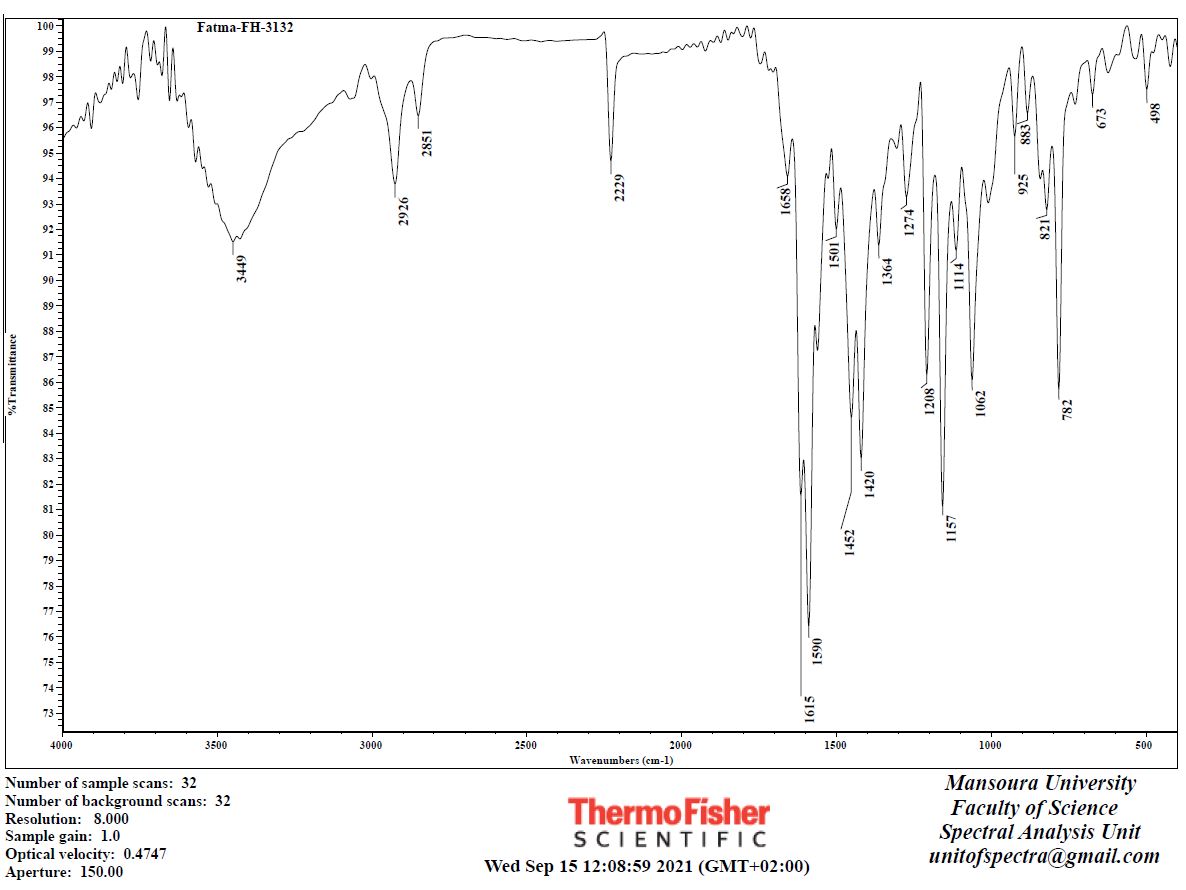**    **Figure (16): IR spectrum of compound 5c** |
| --- |

| 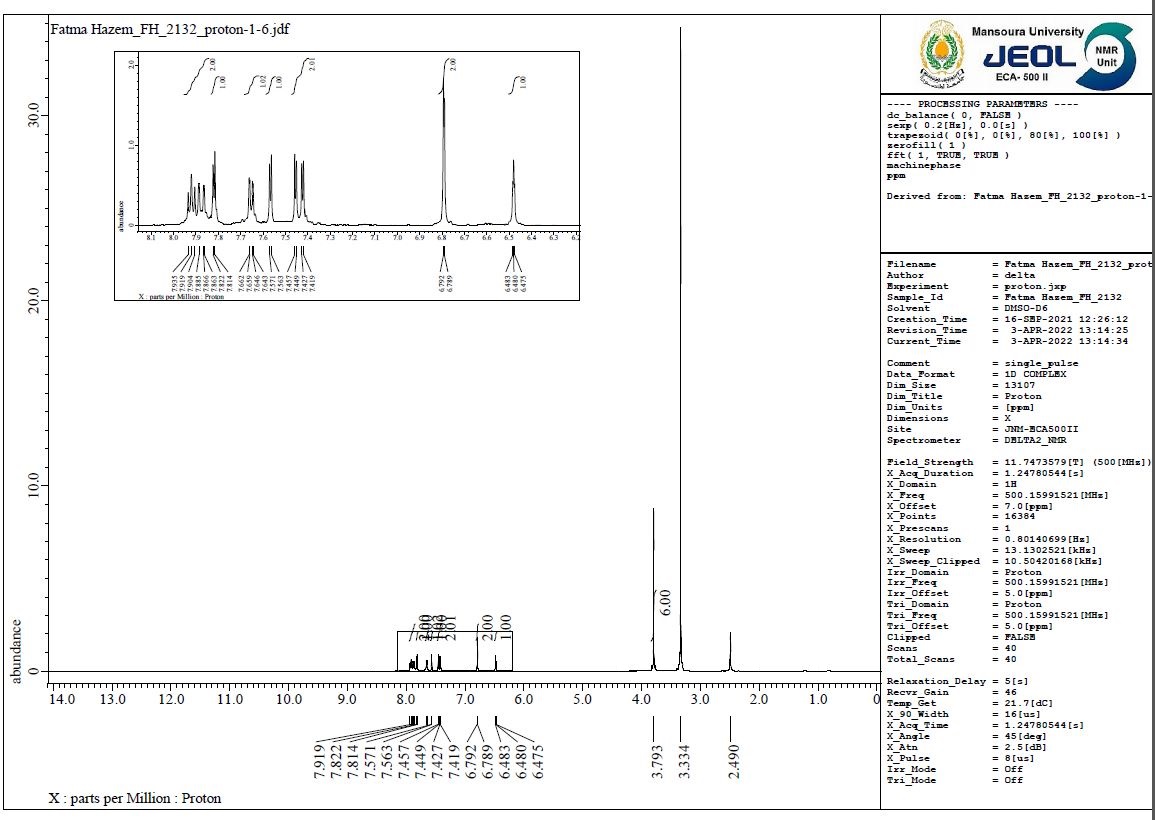    **Figure (17): ^1^H-NMR spectrum of compound 5c** |
| --- |

| **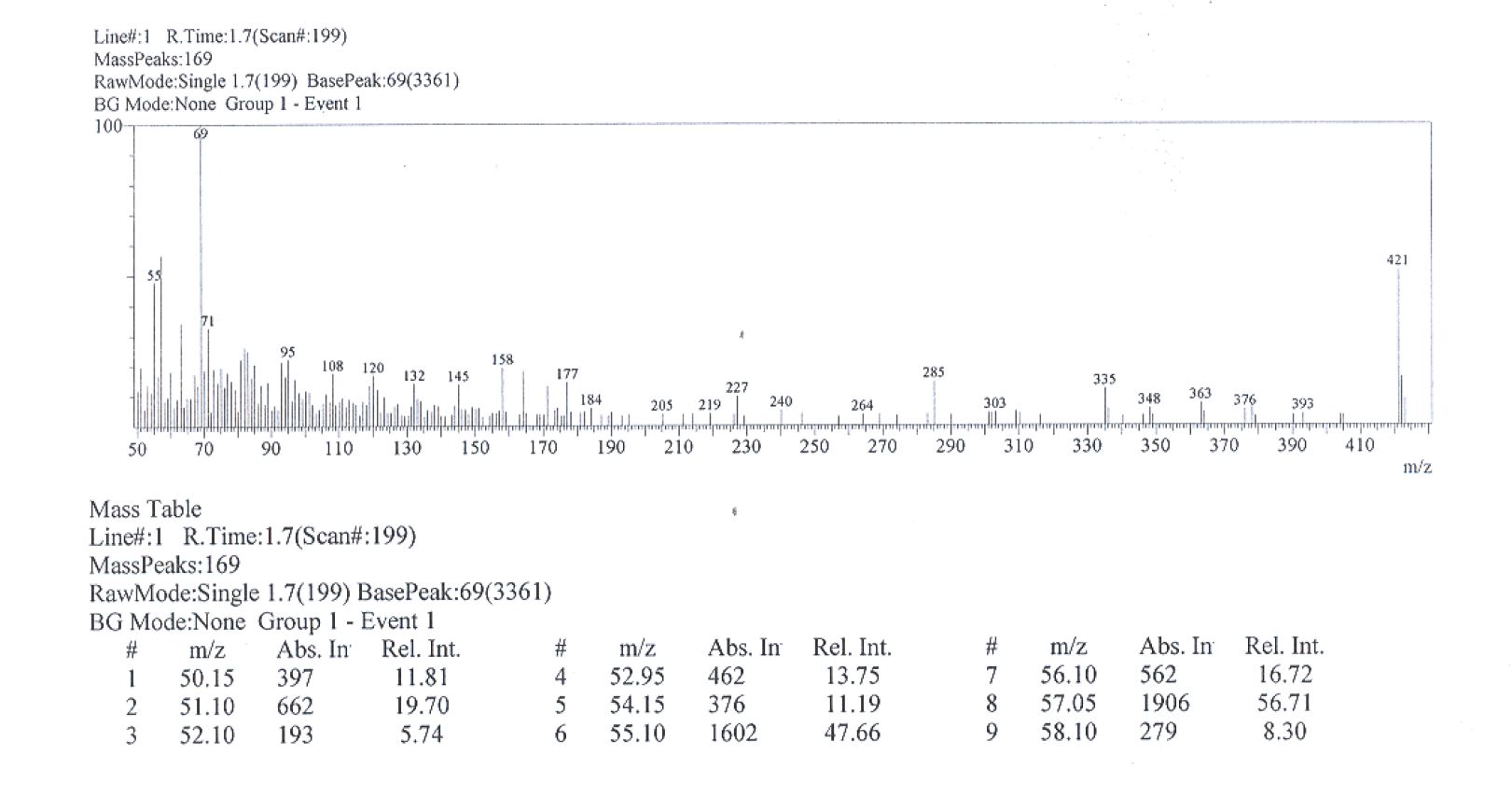**    **Figure (18): Mass spectrum of compound 5c** |
| --- |

**Figure (19): Steady state absorption and fluorescence emission spectra of 3a-c and 5a-c in DMF (2 × 10^–5^ M).**


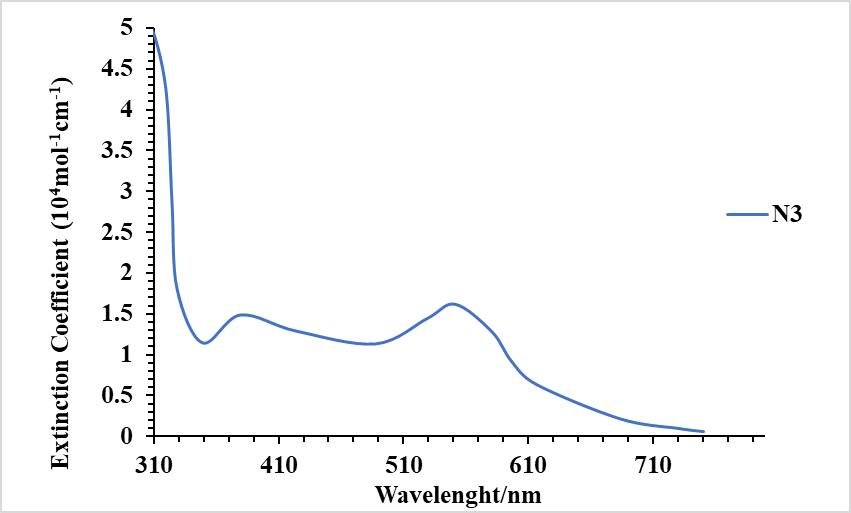


**Figure (20): UV-Vis. absorption of N3.**


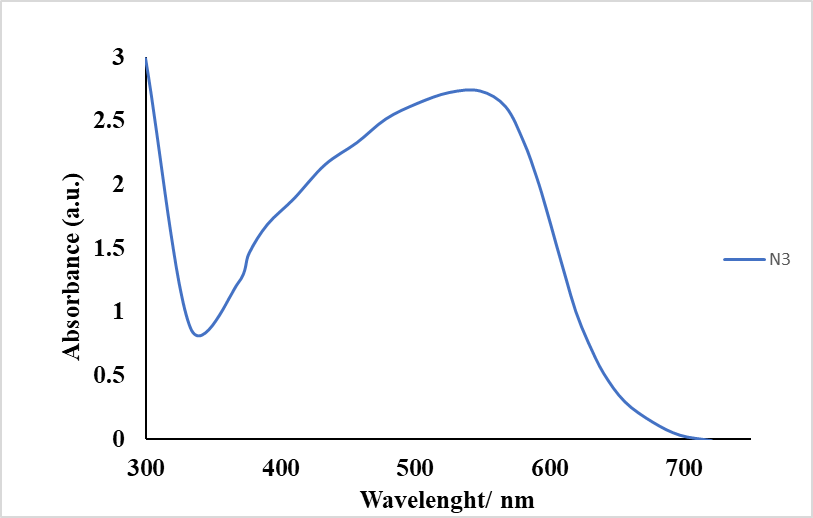
 **Figure (21): UV-Vis. absorption of N3 on TiO_2_.**


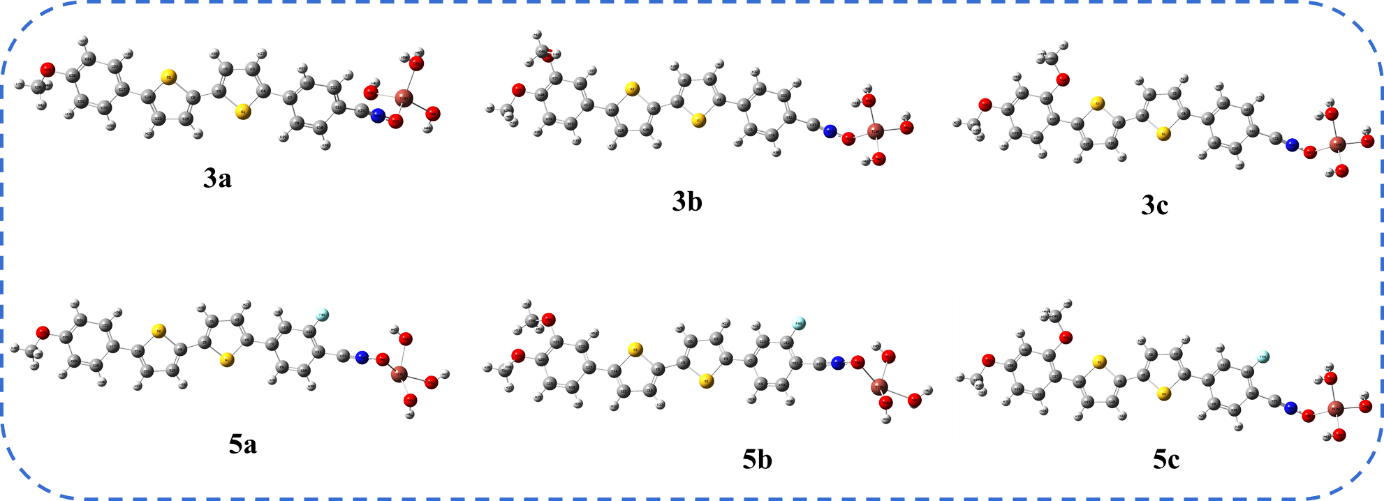


**Figure (22): Interaction of sensitizers co-sensitizers 3a-c and 5a-c with TiO_2_**

| **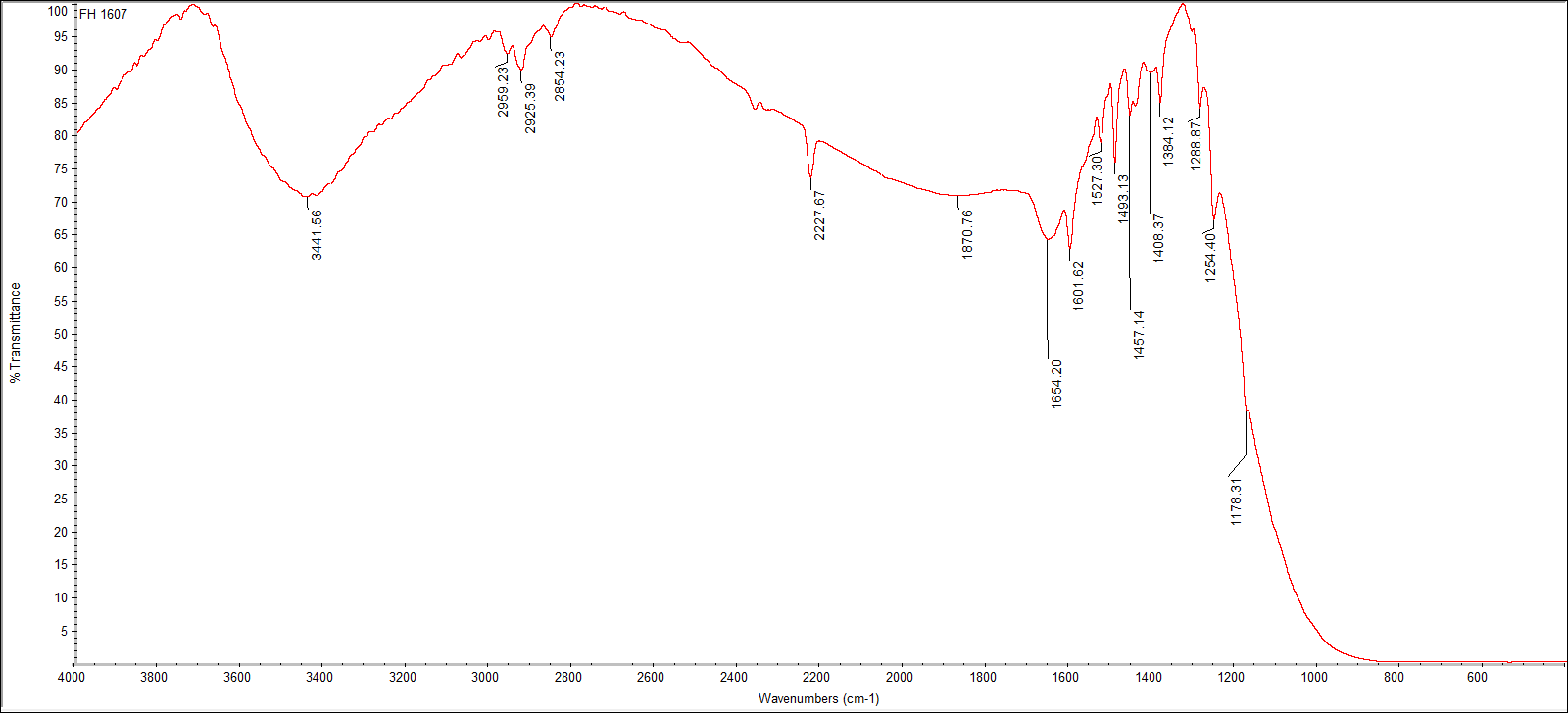**  **Figure (23): IR spectrum of compound 3a over TiO_2_** |
| --- |

| **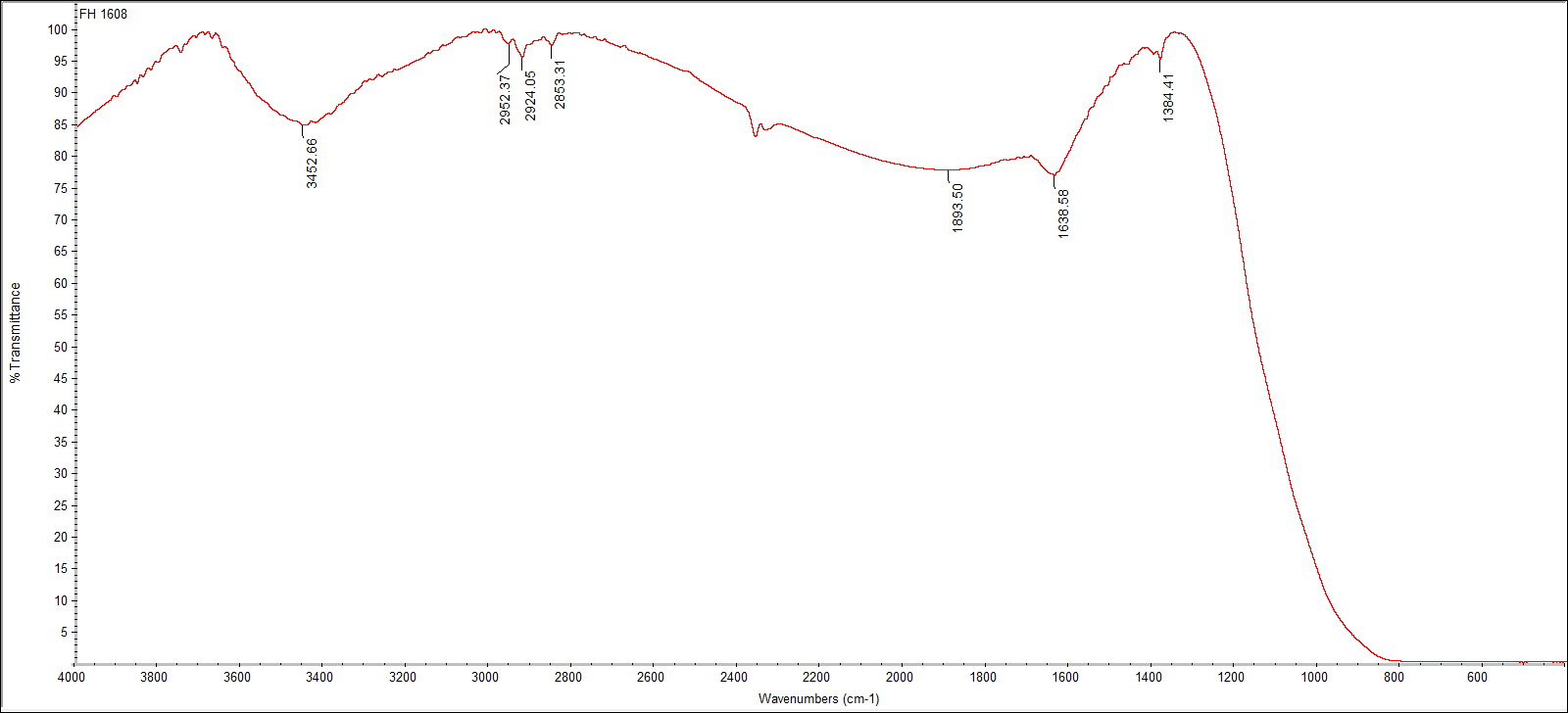**  **Figure (24): IR spectrum of compound 3b over TiO_2_** |
| --- |

| **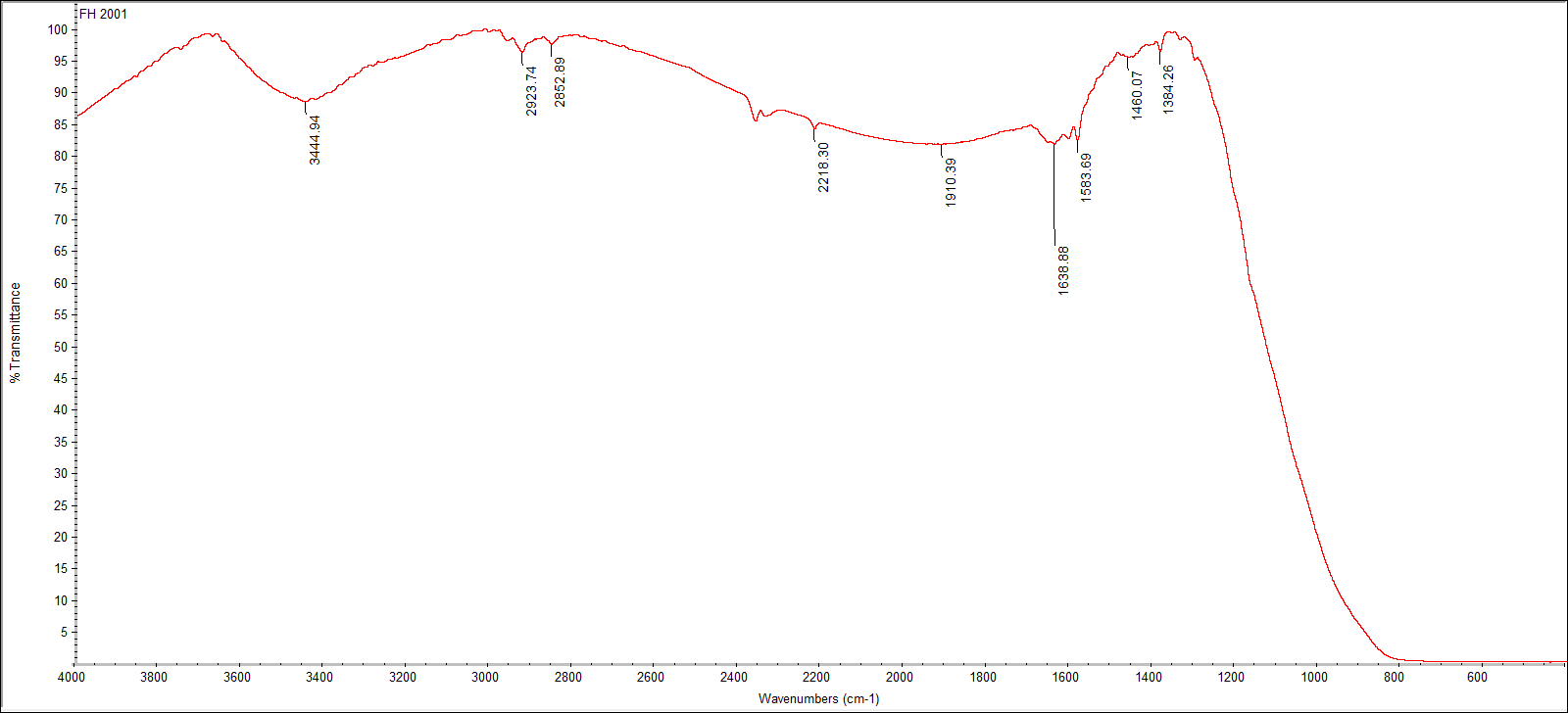**  **Figure (25): IR spectrum of compound 3c over TiO_2_** |
| --- |

| 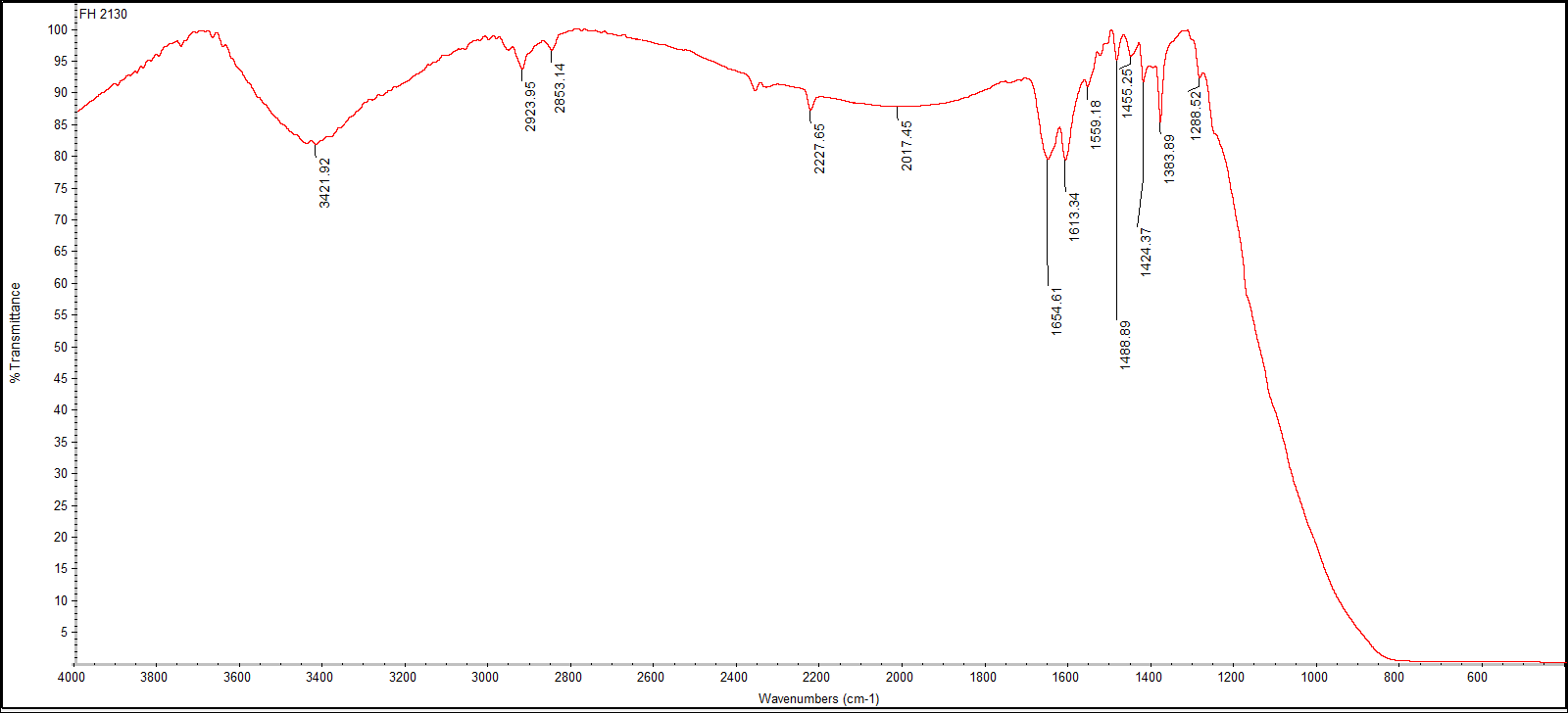  **Figure (26): IR spectrum of compound 5a over TiO_2_** |
| --- |

| **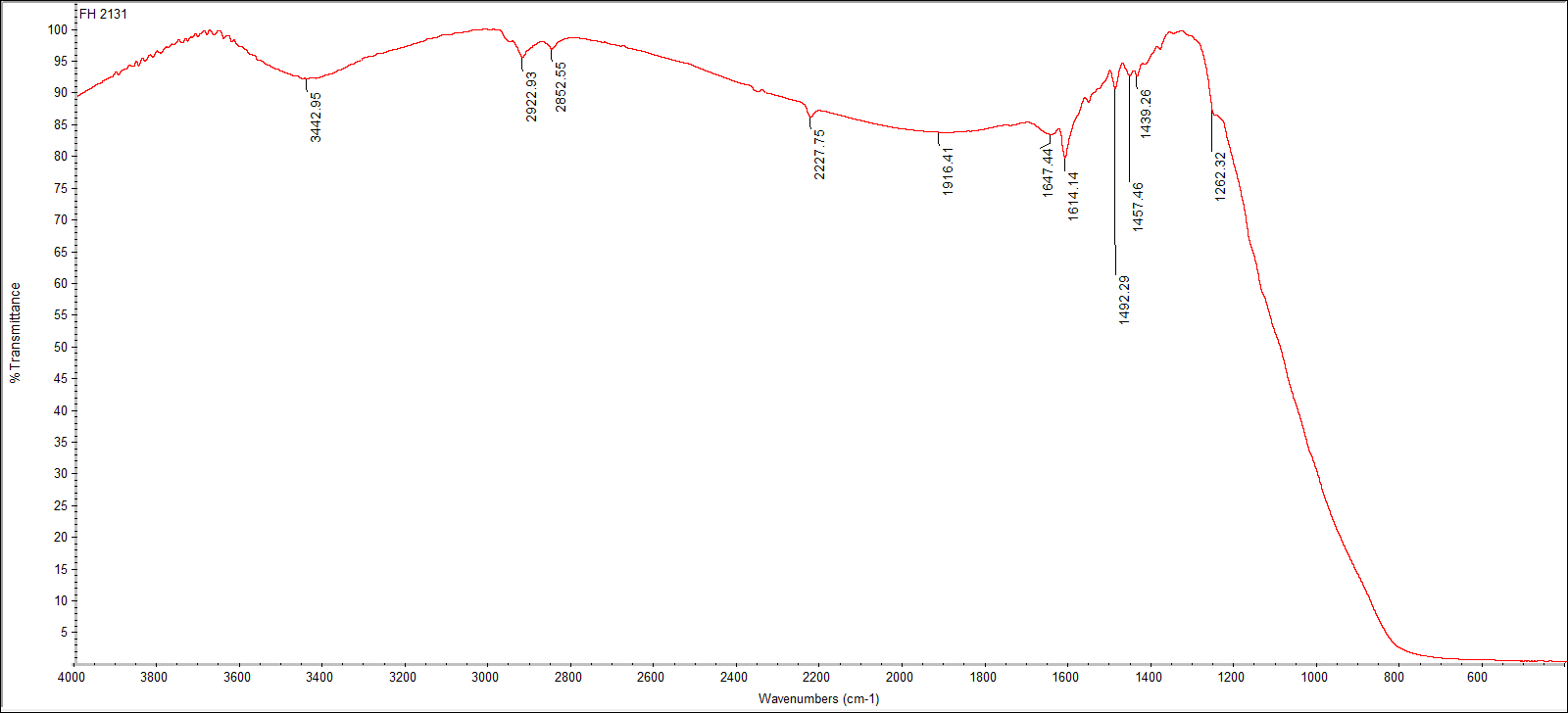**  **Figure (27): IR spectrum of compound 5b over TiO_2_** |
| --- |

| **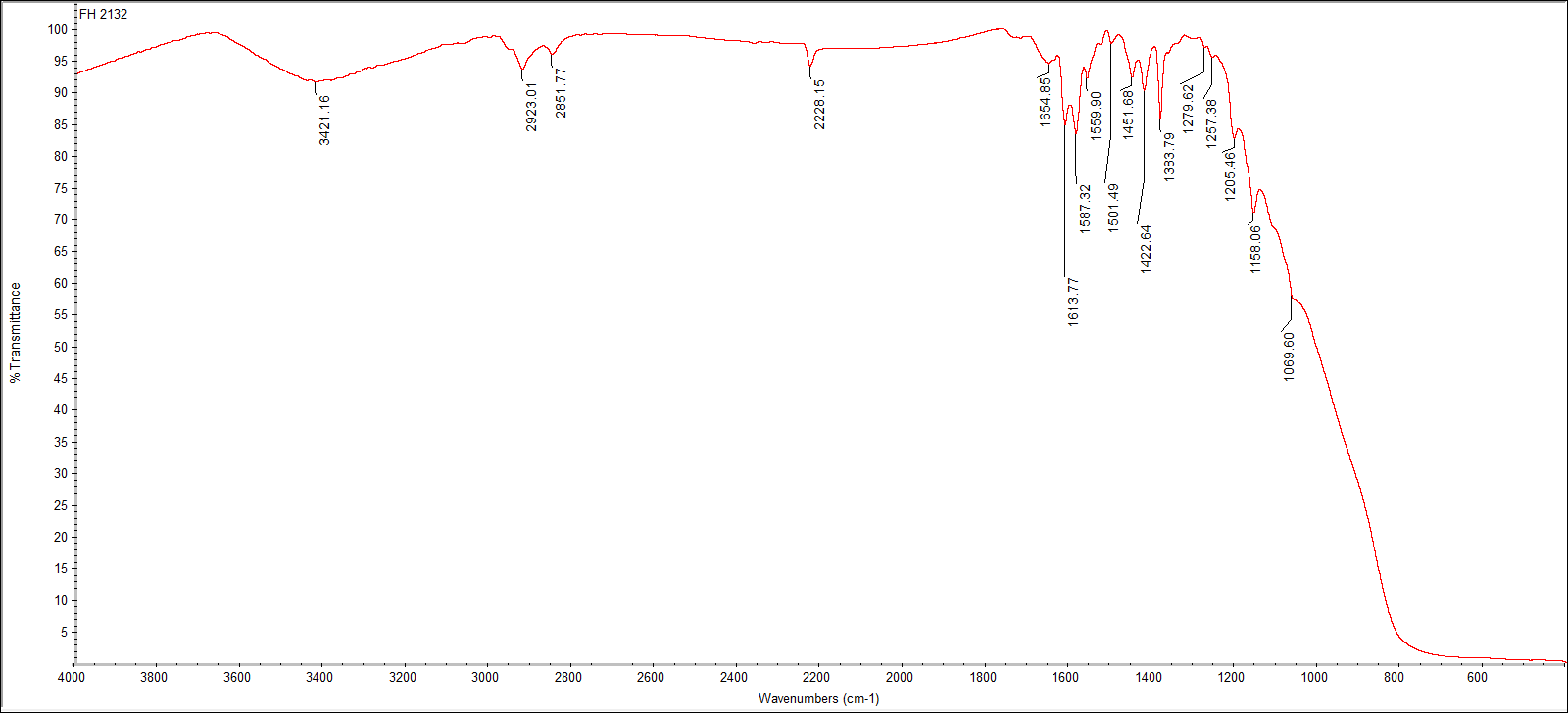**    **Figure (28): IR spectrum of compound 5c over TiO_2_** |
| --- |

**Table 1:** Computed excitation energies, electronic transition configurations and oscillator strengths (*f*) or the optical transitions of the absorption bands for co-sensitizers (3a-c) and (5a-c). (H=HOMO, L=LUMO).

| **Dye** | **Energy (eV)** | **Wavelength**  **(nm)** | **Oscillator Strength (f)** | **Major contribution** | **Minor contributions** | **LHE**  **(1-10*^-f^)*** |
| --- | --- | --- | --- | --- | --- | --- |
| **3a** | **2.6152** | **474** | **1.5628** | **HOMO→LUMO**  **(99.94%)** | - | **0.9730** |
|  | **3.4116** | **363.42** | **0.1027** | **H-1→LUMO**  **(33.51%)** | **HOMO→L+1**  **(36.21%)** |  |
|  | **3.8693** | **320.43** | **0.0040** | **H-2→LUMO**  **(3.14%)** | **HOMO→L+1**  **(16.82%)**  **HOMO→L+2**  **(39.24%)** |  |
| **3b** | **2.5918** | **478.37** | **1.5443** | **HOMO→LUMO**  **(100 %)** | - | **0.9712** |
|  | **3.3655** | **368.40** | **0.1289** | **H-1→LUMO**  **(44.89%)** | **HOMO→L+2**  **(51.83%)** |  |
|  | **3.6848** | **336.48** | **0.0066** | **H-2→LUMO**  **(17.35 %)** | **H-1→LUMO**  **(44.50%)**  **HOMO→L+2**  **(30.53%)**  **HOMO→L+3**  **(2.62%)** |  |
| **3c** | **2.6353** | **470.48** | **1.5744** | **HOMO→LUMO**  **(98.34%)** | - | **0.9733** |
|  | **3.4119** | **363.39** | **0.0771** | **H-1→LUMO**  **(22.80 %)** | **HOMO→L+2**  **(74.54 %)** |  |
|  | **3.8814** | **319.43** | **0.0023** | **H-1→LUMO**  **(10.53 %)** | **HOMO→L+2**  **(64.57 %)**  **HOMO→L+3**  **(5.68 %)** |  |
| **5a** | **2.5956** | **477.67** | **1.6763** | **HOMO→LUMO**  **(99.97 %)** | **-** | **0.9785** |
|  | **3.4216** | **362.36** | **0.1292** | **H-1→LUMO**  **(36.67 %)** | **HOMO→L+1**  **(74.54 %)** |  |
|  | **3.8974** | **318.36** | **0.0047** | **H-3→LUMO**  **(6.64 %)** | **H-1→LUMO**  **(36.73 %)**  **HOMO→L+1**  **(4.68 %)** |  |
| **5b** | **2.6087** | **475.28** | **1.5561** | **HOMO→LUMO**  **(99.97 %)** | - | **0.9722** |
|  | **3.3908** | **365.64** | **0.1197** | **H-1→LUMO**  **(44.79 %)** | **HOMO→L+1**  **(51.41 %)** |  |
|  | **3.6803** | **336.89** | **0.0045** | **H-2→LUMO**  **(26.31 %)** | **H-1→LUMO**  **(42.14 %)** |  |
| **5c** | **2.6175** | **473.67** | **1.5606** | **HOMO→LUMO**  **(98.95 %)** | - | **0.9724** |
|  | **3.4301** | **361.46** | **0.1009** | **H-1→LUMO**  **(25.63 %)** | **HOMO→L+1**  **(71.73 %)** |  |
|  | **3.9045** | **317.54** | **0.0024** | **H-3→LUMO**  **(7.17 %)** | **H-1→LUMO**  **(36.73 %)**  **HOMO→L+2**  **(3.68 %)** |  |

**Table 2.** Compares the performance parameters of **3a-c**, **5a-c** and **N3** with recently reported high-performance sensitizer and co-sensitizer.

| Sensitizers/Co-sensitizers | ***V_OC_* (V)** | ***J_SC_* (mA.cm^-2^)** | ***FF* (%)** | ***η (%)*** | ***References*** |
| --- | --- | --- | --- | --- | --- |
| **3a+ N3** | **0.511** | **15.67** | **59.16** | **4.73** | This work |
| **3b+ N3** | **0.519** | **15.38** | **58.13** | **4.64** | This work |
| **3c+ N3** | **0.499** | **14.13** | **58.55** | **4.13** | This work |
| **5a+ N3** | **0.676** | **18.14** | **60.54** | **7.42** | This work |
| **5b+ N3** | **0.565** | **17.17** | **60.01** | **5.82** | This work |
| **5c+ N3** | **0.637** | **17.42** | **59.18** | **6.57** | This work |
| **GA1+N3** | **0.71** | **20.72** | **62** | **9.12** | *https://doi.org/10.1016/j.optmat.2023.114031* |
| **GA1+N3** | **0.68** | **19.56** | **61** | **8.11** | *https://doi.org/10.1016/j.optmat.2023.114031* |
| **GA3+N3** | **0.65** | **17.81** | **59** | **6.83** | *https://doi.org/10.1016/j.optmat.2023.114031* |
| **GA4+N3** | **0.66** | **18.66** | **61** | **7.51** | *https://doi.org/10.1016/j.optmat.2023.114031* |
| **GA5+N3** | **0.62** | **16.99** | **59** | **6.22** | *https://doi.org/10.1016/j.optmat.2023.114031* |
| **A1 +N3** | **0.612** | **13.99** | **61.09** | **5.23** | *https://doi.org/10.1016/j.solener.2018.09.071* |
| **A2 +N3** | **0.639** | **15.36** | **63.15** | **6.20** | *https://doi.org/10.1016/j.solener.2018.09.071* |
| **A3 +N3** | **0.608** | **14.19** | **59.51** | **5.13** | *https://doi.org/10.1016/j.solener.2018.09.071* |
| **A4+N3** | **0.625** | **15.05** | **63.32** | **5.95** | *https://doi.org/10.1016/j.solener.2018.09.071* |
| **A5+N3** | **0.604** | **14.40** | **61.98** | **5.39** | *https://doi.org/10.1016/j.solener.2018.09.071* |
| **RK-1+N3** | **0.748** | **14.423** | **59.410** | **6.40** | ***https://doi.org/10.1039/C5RA26577K*** |
| **F1+N719** | **0.733** | **23.28** | **58.4** | **9.97** | *https://doi.org/10.1007/s10854-022-08470-9* |
| **F2+N719** | **0.609** | **19.96** | **57.7** | **6.95** | *https://doi.org/10.1007/s10854-022-08470-9* |
| **F3+N719** | **0.637** | **21.31** | **58.10** | **7.88** | *https://doi.org/10.1007/s10854-022-08470-9* |
| **F1** | **0.654** | **10.66** | **64.30** | **4.41** | *https://doi.org/10.1007/s10854-022-08470-9* |
| **F2** | **0.501** | **6.30** | **61.50** | **1.98** | *https://doi.org/10.1007/s10854-022-08470-9* |
| **F3** | **0.557** | **6.99** | **62.40** | **2.43** | *https://doi.org/10.1007/s10854-022-08470-9* |

**4. Fabrication of dye-sensitized solar cell**

Photovoltaic measurements were made on sandwich cells, which were prepared using TiO_2_ coated working electrodes and platinum coated counter electrodes and were sealed using a 40 μm Syrlyn spacer through heating of the polymer frame. The redox electrolyte (Solaronix, Iodolyte HI-30) consisted of a solution of 0.6 M DMPII, 0.05 M I_2_, 0.1 M LiI and 0.5 M TBP in acetonitrile.

**4.1. Cell fabrication process including the co-sensitization process as no cell fabrication process**

Fabrication of dye-sensitized solar cells (DSSC) to prepare the working FTO electrodes, the FTO coated glass substrates was cleaned in an order of detergent solution with sonication by an ultrasonic bath for 30 min and then thoroughly rinsed with deionized (DI) water and ethanolic solution. TiO_2_ paste was prepared by mixing grounding the TiO_2_ powder with distilled water, acetic acid, acetylacetone and polyethylene glycol (PEG). The slurry was ultrasonicated for 50 min and was stirred at 1100 rpm for 60 min. Ultrasonication and stirring were repeated 4 times to get a consistent viscous paste of TiO2. The TiO_2_ films were made by spreading TiO2 paste between two Scotch tapes on FTO by the Doctor-blade method. When the paste becomes dry, the two tapes are removed and the TiO_2_ mold is dried at 500 C for 50 min. TheTiO_2_-layer with a thickness of 10–12 lm is controlled by the scotch tape. The dyes sensitized TiO_2_/FTO photoelectrodes were prepared through soaking the TiO_2_/FTO films in a 0.5 mM dye solution (ethanol) for 1 day. Thin layer of Pt-paste (Solaronix, Platisol T/SP) on TCO was printed and the printed electrodes were then cured at 450 °C for 10 min. The counter electrode is fabricated by the drop-casting method of platinum paste (PT1, Dyesol) onto FTO glass plates. After that, two holes are drilled on the counter electrode to facilitate electrolyte injection. The iodide/triiodide (I^-^ /I^-^_3_) was used as the electrolyte solution. The resulting counter electrodes and the working TiO_2_/FTO electrodes were sealed using a thick Surly. By injecting iodide/triiodide (I^-^ /I^-^_3_) electrolyte into the cells through one of the two small holes in the counter electrodes we could prepare TiO_2_-based DSSCs with a 20 mm^2^ active area. The dye solutions of the main sensitizer N3 (0.2 mM) were prepared in 1:1:1 mixture of acetonitrile, tert-butyl alcohol and dimethyl sulfoxide (DMSO), 0.2 mM of co-sensitizer (**3a-c** and **5a-c**), were prepared. A mixture of 1:1 acetonitrile, tert-butyl alcohol was used as a solvent. For co-sensitization, **3a-c** and **5a-c** with N719 (0.2mM **3a-c** and **5a-c** + 0.2mM N3) were dissolver in 1:1:1 mixtures of the same solvent used later. The electrodes were immersed in the dye solutions and then kept at 25 ° C for 20 h to adsorb the dye onto the TiO_2_ surface. The performance of the dye-sensitized solar cells was characterized by photovoltaic measurements of sealed cells were made by illuminating the cell through the conducting glass from the anode side with a solar simulator at AM 1.5 illuminations (light intensity: 100 Mw.cm^-2^).

**5. Photovoltaic measurements**

Photovoltaic measurements of sealed cells were made by illuminating the cell through the conducting glass from the anode side with a solar simulator at AM 1.5 illuminations (light intensity: 100 mW cm^−2^).

**6. Electrochemical impedance spectroscopy (EIS)**

The electrochemical impedance spectra were measured with an impedance analyzer potentiostat (Bio-Logic) under illumination using a solar simulator. The electrical impedance spectra were fitted using Z-Fit software (Bio-Logic).

**7. Molecular Modeling**

Equilibrium molecular geometries of **3a-c** and **5a-c** calculated using the Becke's three parameter hybrid functional, Lee-Yang-Parr's gradient corrected correlation functional (B3LYP) and (6-311G (d, p)) [2, 3, 4, 5]. The geometry optimization calculations were followed by energy calculations using time-dependent density functional theory (TD-DFT) utilizing the energy, functional B3LYP and the basis set 6-311G (d, p), implemented in Gaussian 09.

**References**

[1] Yokooji, A., Satoh, T., Miura, M. and Nomura, M., 2004. Synthesis of 5, 5′-diarylated 2, 2′-bithiophenes via palladium-catalyzed arylation reactions. Tetrahedron, 60(32), pp.6757-6763.

[2] Melikian, G., Rouessac, F., & Alexandre, C. (1993). A convenient synthesis of substituted 3-pyrrolin-2-ones from α-cetols, *Synthetic Communications,* 23, 2631-2638, https://doi.org/10.1080/00397919308013792

[3] Becke, A.D. (1988). Density-functional exchange-energy approximation with correct asymptotic behavior, *Physical Review A,* *38*, 3098-3100, https://doi.org/10.1103/PhysRevA.38.3098.

[4] Lee, C. T., Yang, W.T., & Parr, R.G. (1988). Development of the Colle-Salvetti correlation-energy formula into a functional of the electron density. *Physical Review B, 37,* 785-789 https://doi.org/10.1103/PhysRevB.37.785

[5] Godbout, N., Salahub, D. R., Andzelm, J., & Wimmer, E. (1992). Optimization of Gaussian-type basis sets for local spin density functional calculations. Part 1. Boron through neon, optimization technique and validation. *Canadian Journal of Chemistry,* 70, 560-571, https://doi.org/10.1139/v92-079@cjc-uc-0101.
